# Mechanistic Insights into the Early-Stage Crystallization and Nanophase Formation of Metastable Light Rare-Earth Carbonates

**DOI:** 10.1021/acs.cgd.4c01168

**Published:** 2025-02-08

**Authors:** Luca Terribili, Adrienn Maria Szucs, Melanie Maddin, Kristina Petra Zubovic, Remi Rateau, Juan Diego Rodriguez-Blanco

**Affiliations:** † Department of Geology, School of Natural Sciences, Trinity College Dublin, College Green, Dublin D02PN40, Ireland; ‡ iCRAG, Department of Geology, School of Natural Sciences, Trinity College Dublin, College Green, Dublin D02PN40, Ireland

## Abstract

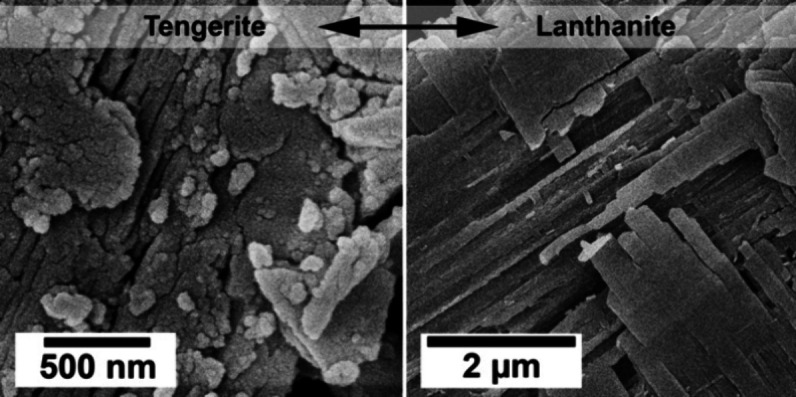

Rare-earth element (REE) carbonates play a crucial role in geochemistry due to their prevalence in carbonatite ore deposits, which are extensively mined globally for REE extraction. Two primary mineral groups of interest are lanthanites (REE_2_(CO_3_)_3_·8H_2_O) and bastnäsites (REECO_3_(OH,F)), typically enriched in light REE. This study aims to elucidate the mechanisms and kinetics of La-, Ce-, Pr-, and Nd-carbonate crystallization reactions at the earliest stages. REE-carbonates were synthesized through homogeneous crystallization by combining CO_3_^2–^ and REE-bearing solutions at temperatures ranging from near-ambient to low hydrothermal conditions (5–80 °C). The crystallization processes were monitored in situ and in real-time using UV–vis spectrophotometry and synchrotron-based wide-angle X-ray scattering (WAXS). The characterization and quantification of the newly formed phases were conducted using a combination of conventional powder X-ray diffraction, high-resolution scanning electron microscopy with energy-dispersive spectroscopy and Fourier transform infrared spectroscopy. Our findings reveal a complex, multistep crystallization pathway specific to each REE, influenced by factors such as temperature, solution concentration and ratio, phase stability, and REE ionic potential. Additionally, the REE-carbonate crystallization pathways align with a progressive dehydration sequence involving multiple intermediate nanophases and reversible reactions. Notably, a reversible reaction between lanthanite and nanotengerite was observed at ambient temperature, involving structural rearrangements and hydration-dehydration processes. Our findings emphasize the importance of nanophase formation during the initial stages of REE-carbonate crystallization, with implications for the development of more efficient REE extraction methods.

## Abstract 2

This study reveals complex, multistep pathways for light rare earth carbonate crystallization, emphasizing the role of nanophases and reversible reactions at early stages.

## Introduction

The rare-earth elements (REE) are never found in nature as native metals such as gold or copper. Instead, they occur in a variety of minerals comprising carbonates, phosphates, oxides, silicates, and halides.^1−6^ REE-carbonates are of primary importance in geochemistry^7^ as they are commonly mined from carbonatite deposits of which a well-known example is Bayan Obo (China), the largest known in the world.^3,5,6^ Two primary representative REE-carbonate minerals groups are found in these deposits: bastnäsites, REECO_3_(OH,F), and lanthanites, REE_2_(CO_3_)_3_·8H_2_O.^8,9^ These are two REE-carbonate groups, typically dominated by La, Ce, Pr, and Nd as major elements,^2,8,10^ which are recognized for their LREE selectivity and often exhibiting enrichment in the following sequence: Ce > La > Nd.^11^ Experimental research has demonstrated that at low hydrothermal conditions, REE-carbonates crystallization is consistent with the progressive dehydration sequence REE_2_(CO_3_)_3_·8H_2_O (lanthanite, orthorhombic) → REECO_3_OH (kozoite, orthorhombic) → REECO_3_OH (hydroxylbastnäsite, hexagonal) whereas cerianite (CeO_2_, cubic) can also crystallize in Ce-rich samples.^12−18^

Focusing on these two REE-carbonate groups, bastnäsite (Hexagonal, *P*6̅*c*2 space group) is one of the most important mineral groups as it is the most thermodynamically stable REE-carbonate and a key source of REE for industry applications.^1−6^ It occurs naturally as hydroxylbastnäsite (REECO_3_OH) and fluorobastnäsite (REECO_3_F), with the latter being the predominant member in nature.^10,11,19,20^

Kozoite and lanthanite are less abundant compared to bastnäsite. Kozoite (Orthorhombic, Pmcn space group) is a member of the ancylite supergroup which has a large chemical variability^14^ and is a polymorph of hydroxylbastnäsite.^21^ It was first reported by Miyawaki et al.^22^ and it has been found in association with an alkali olivine basalt and in pegmatite deposits.^22^ This mineral forms very commonly as a transient metastable phase during the experimental synthesis of REE-carbonates at a wide range of temperatures and transforms to hydroxylbastnasite via a dissolution-recrystallization reaction,^12−18^ similar to the occurrence of vaterite during the synthesis of calcite.^23−25^

Lanthanite is a group of REE hydrated carbonates that were originally reported coating REE-silicates like cerite (REE_9_(Fe^3+^,Mg)(SiO_4_)_6_(SiO_3_OH)(OH)_3_) at the Bastnäs mine in Sweden^26,27^ and its composition was first determined by Blake^28^ and Genth.^29^ Lanthanites are orthorhombic (Pbnb space group) (e.g., refs (30−32)) and consist of layers of 10-fold coordinated REE-O polyhedra and CO_3_ groups connected by hydrogen bonds to interlayer water molecules, forming a highly hydrated mineral structure.^8,33^ Previous studies on lanthanites primarily focused on analyzing natural samples sourced from various ores worldwide to determine mineral structure and chemical composition (e.g., refs (27,30,32−36)). Other studies focused on the synthesis of different REE lanthanites for their characterization and study of their solubilities,^37−43^ thermochemical properties,^9,19,44^ kinetics and mechanisms of crystallization from solution^8,13,45^ or during mineral replacement processes.^14−17^

Understanding the complex crystallization pathways and mechanisms involved in the formation of these REE-bearing minerals is of great importance to designing better sequential extraction methods via fractional crystallization (e.g., refs (46−48)). Besides, there is still a considerable gap in the knowledge of the earliest stages of crystallization of REE-carbonates. It is known that these reactions are temperature-dependent and their number of intermediate steps, among the other factors, is primarily influenced by the temperature, ionic radii of REE, and their concentration in solution^13−17^ thus suggesting potential complexity beyond initial expectations.

In this study, we investigate the formation processes of different metastable REE-carbonates from amorphous precursors across a temperature range from ambient to low hydrothermal conditions (5–80 °C). By following in situ and real-time the crystallization of REE-carbonates through the use of UV–vis spectrophotometry and synchrotron-based diffraction and by characterizing the solids using powder X-ray diffraction (XRD), Fourier transformed infrared spectroscopy (FTIR), and high-resolution microscopy (SEM-EDS), we aim to shed light on the early stages of REE-carbonates crystallization reactions.

## Materials and Methods

To study the early-stage kinetics, mechanisms, and pathways of REE-carbonate crystallization, a series of experiments were carried out at various temperatures ranging from ambient to low hydrothermal (5–80 °C) conditions. These experiments were performed by homogeneously mixing 40 mL of 50 mM Na_2_CO_3_ with 40 mL of 50 mM La-, Ce-, Pr-, or Nd-bearing aqueous solutions in glass reactors. The solutions were preheated or precooled to the desired specific temperatures (5, 21, 35, 50, and 80 °C). The reactors were then sealed and placed in a preheated oven, a precooled fridge, or left at ambient temperature under static conditions. All solutions were prepared using reagent-grade Na_2_CO_3_ or REE nitrate hexahydrates (La(NO_3_)_3_·6H_2_O, Ce(NO_3_)_3_·6H_2_O, Pr(NO_3_)_3_·6H_2_O; Sigma-Aldrich 99.99% trace metals basis) and pure deionized (Milli-Q) water.

For the ambient temperature experiments, which were very long and required a good number of sample points, two exact replica experiments were carried out. This procedure allowed us to get some advantages: to increase the maximum number of sample points, to have a “control” experiment to validate our data, and not to deplete all the solutions in the reactors.

The crystallization reactions were monitored by collecting 5 mL of suspensions from the reactors at specific time intervals using a pipet. Following the methods described in Rodriguez-Blanco et al.,^49^ Tobler et al.,^50^ Terribili et al.^51^ and Terribili et al.,^52^ the samples were promptly filtered using a vacuum filtration system with 0.2 μm polycarbonate filters, rinsed with isopropanol to prevent potential recrystallization of other solids from residual interstitial water and immediately air-dried.

Identification and quantification of the newly crystallized solid phases were carried out with powder X-ray diffraction (XRD). Analyses were performed with a Bruker D5000 powder X-ray diffractometer (Cu Kα radiation, 0.02 step^–1^ from 5 to 70° in 2θ at 0.2° min^–1^) located at Trinity Technology and Enterprise Centre (Dublin). The crystalline phases in the samples were identified with Bruker DIFFRAC.EVA software jointly with the ICDD Powder Data File (PDF-4; The International Centre for Diffraction Data). Pattern-matching refinement and quantification of crystalline phases were carried out with the Rietveld refinement software TOPAS.^53^ The crystallite size and the unit cell parameters were refined for all the minerals analyzed, while the preferred orientation was refined only for lanthanite in the (002) plane.

Infrared spectra of the selected samples were acquired by using a Nicolet Summit Fourier-transform infrared (FTIR) spectrometer with an Everest Diamond ATR accessory, with a wavelength range between 4000 and 650 cm^–1^, and a resolution of 4 cm^–1^. The OMNIC Paradigm Desktop Software was used for data analysis and characterization.

Images of solids were obtained using scanning electron microscopy equipped with energy dispersive spectroscopy (SEM-EDS) to study potential changes in the morphology and size of the crystalline phases. Samples were prepared by placing them on mounts and coating them with Au for imaging. SEM-EDS analyses were conducted using a Tescan TIGER MIRA3 FEG-SEM operating under high vacuum conditions and equipped with two Oxford Instruments X-Max 150 mm^2^ energy dispersive spectroscopy (EDS) detectors running Oxford Instruments AZtec analysis software. The analyses were carried out using a beam current of 300 pA and an accelerating voltage of either 5 or 10 kV, for detailed imaging, or 20 kV, for EDS analysis at the iCRAG Lab at Trinity College Dublin. The particle size distribution analysis of the samples was carried out with ImageJ Software.^54−56^

Time-resolved UV–vis spectrophotometry was used for monitoring the crystallization reactions of La-, Ce-, Pr-, and Nd- lanthanites in situ and real-time at different temperatures between 19 and 55 °C). The selection of temperatures was based on XRD analyses to ensure this mineral was the primary and sole product of the crystallization reactions. Following the methods of Tobler et al.^50,57,58^ and Terribili et al.^51^ the change in solution absorbance (turbidity) was examined by mixing equal volumes (1 mL) of 10 mM La-, Ce-, Pr- or Nd-bearing solutions and 10 mM of Na_2_CO_3_ solutions in a 5 mL cuvette under gently and continuing stirring conditions. The UV–vis spectrophotometer (Ocean Optics) was set to measure the absorbance at 450 nm wavelength at time intervals of 1 s. The solid samples were filtered according to the aforementioned method and then analyzed with the XRD in order to verify the nature of the newly crystallized phases. In these real-time UV–vis experiments, we consider that nucleation begins together with the increase in absorbance, although there is a possibility that a very tiny fraction of the particles may crystallize just before the initial rise in absorbance. Any error resulting from this assumption is expected to be proportionally consistent across all experiments regardless of the aqueous chemistry. In the same way, the maximum absorbance value is going to be considered to correspond to the moment when the primary crystallization process is completed.^51^

The data set obtained from the UV–vis experiments was used to calculate the reaction rates and to get the apparent activation energies of crystallization (*E*_a(cryst.)_) and nucleation (*E*_a(nucl.)_). For this purpose, the data was fitted to a JMAK (Johnson-Mehl-Avrami–Kolmogorov) model, based on the Avrami equation^59^:
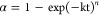
1where *k* is a rate constant, *t* is time, α is the fraction crystallized, and *n* is a constant that depends on the crystallization mechanism. Rewriting the Avrami Equation gives
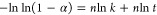
2

A reaction with kinetics that fit this equation results in a straight line when −ln ln(1 – *y*) is plotted against ln *t*.^52,60,61^ The value of parameter n is given by the value of the line slope, and it can be used to compare reaction mechanisms. Parallel lines indicate a constant value of *n*, indicating a similar reaction mechanism. The intercept on the *y-*axis gives the value of *n* ln *k*, by which the *k* value can be determined.^15,52^ Even if this model was originally applied to solid-state transformations and phase transitions in metals and alloys, it has also been successfully used to describe mineral-water interaction reactions (e.g., refs (52,62−65)).

The apparent activation energy of crystallization (*E*_a(cryst.)_) and the apparent activation energy for nucleation (*E*_a(nucl.)_) were calculated using the induction times (*t*_0_) and rate constants (*k*) as a function of temperature using an Arrhenius approach.^23,66,67^
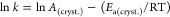
3
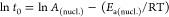
4

In these equations, *A* is a pre-exponential factor (s^–1^), *E*_a(cryst.)_ is the activation energy for crystallization (kJ mol^–1^), *E*_a(nucl.)_ is the activation energy for nucleation (kJ mol^–1^), *R* is the gas constant (8.314 J mol^–1^ K^–1^), and *T* is the temperature expressed in K.

*E*_a(cryst)_ is an “apparent” activation energy because it represents contributions from all the many processes occurring in the reaction (e.g., dissolution, aggregation) and not just the potential barrier for a single reaction.^52,66−68^ The *t*_0_ values were determined through the analysis of the reaction curves obtained as a result of the different UV–vis experiments.

### Synchrotron Experiments

The early stages of crystallization of Ce-carbonates were also investigated in situ and in a time-resolved mode by using WAXS at beamline I22 at the Diamond Light Source Ltd. (UK). For this experiment, equal volumes of 50 mM Ce(NO_3_)_3_ and 50 mM Na_2_CO_3_ solutions were injected into a 3-neck reactor flask (250 mL) using a remotely controlled peristaltic pump (Gilson Mini Puls 3) at 200 mL/min. The resulting mixed liquid/suspension was continuously stirred at 300 rpm and circulated at a flow rate of 50 mL/min through a flow-through cell containing a borosilicate glass capillary (*d* = 1.5 mm) that was aligned perpendicular to the X-ray beam. The reactor was placed in a precooled oil bath with an initial temperature of 18 °C. Wide-angle X-ray scattering (WAXS) data were acquired by using a monochromatic X-ray beam at 16 keV. Two-dimensional WAXS intensities were collected at intervals of 30 s with a Decris Pilatus 300k detector (2D large-area pixel array detector),^69^ calibrated with synthetic and highly crystalline silicon (NIST SRM 640C). This silicon standard was used to determine the areas of selected Bragg peaks. The WAXS detector covered the *q*-range of 23.9 < *q* < 38.7 nm^–1^. All recorded patterns were normalized and background-corrected using water.

### Lanthanite Dehydration and Rehydration Experiments

Dehydration and hydration experiments were carried out on a (La)-lanthanite sample synthesized through homogeneous mixing crystallization at ambient temperature (21 °C). Part of the solid sample obtained in this way (2 g) was placed in a 50 mL porcelain crucible with a lid and inserted in an oven preheated at a constant temperature of 80 °C. Samples of 0.2 g each were then collected at increasing time intervals and analyzed using conventional XRD. A portion of the final sample, obtained after full completion of the dehydration process, was then used to investigate potential rehydration processes of the crystalline phase present in it. For this purpose, 0.3 g of samples were placed in 15 mL glass reactors filled with Milli-Q water and left in static conditions at 21 °C. Samples were then collected at increasing time intervals and subsequently analyzed using conventional XRD.

### PHREEQC Calculations

The saturation indices (SI) of REE-bearing carbonates during the equilibration of REE-bearing aqueous solutions with respect to lanthanite, kozoite, hydroxylbastnäsite, and cerianite were calculated through the hydrogeochemical code PHREEQC^70^ using the LLNL database.^71^ The solubility products of REE-carbonates are not well-known and data in the existing literature are scarce and not available for all the REE. For our calculations, the solubility products of REE-carbonates determined by Essington and Mattigod^72^ and Voigt et al.^73^ were used.

## Results

### Homogeneous Crystallization Experiments

The homogeneous crystallization experiments revealed that the early stages of REE-carbonate crystallization involved the formation of metastable phases and that their crystallization kinetics and pathways depended on temperature and the specific REE used (Tables 1–3). The combined use of UV–vis spectrophotometry, conventional and synchrotron-based diffraction, infrared spectroscopy, and SEM-EDS microscopy allowed for in situ and real-time monitoring of REE-carbonates crystallization, as well as the identification and quantification of the newly formed phases.

**Table 1:** Quantification of Crystalline Phases during the La-Carbonate Crystallization Experiments at Different Temperatures

La						
*T* (°C)	time (h)	lanthanite wt %	kozoite wt %	tengerite wt %	hydroxylbastnäsite wt %	reactor(s)
5	4	100	/	/	/	1
	7	100	/	/	/	1
	25	100	/	/	/	1
	27	100	/	/	/	1
	32	100	/	/	/	1
	49	100	/	/	/	1
	75	100	/	/	/	1
	147	100	/	/	/	1
21	1.5	100	/	/	/	1
	2.5	36	/	64	/	1
	4	61	/	39	/	1
	6	100	/	/	/	1
	8	100	/	/	/	1,2
	25	100	/	/	/	1
	28	100	/	/	/	1
	32	100	/	/	/	1
	48	100	/	/	/	1
	74.5	100	/	/	/	1
	144	100	/	/	/	1,2
	148	100	/	/	/	2
	168	100	/	/	/	2
	337	100	/	/	/	2
	505	100	/	/	/	2
	671	100	/	/	/	2
	815	92	8	/	/	2
35	24	82	18	/	/	1
	50	78	22	/	/	1
	192	46	54	/	/	1
	360	/	100	/	/	1
	526	/	100	/	/	1
	670	/	100	/	/	1
	696	/	100	/	/	1
	865	/	100	/	/	1
	1201	/	100	/	/	1
50	0.5	100	/	/	/	1
	1.5	100	/	/	/	1
	3	42	58	/	/	1
	5	25	75	/	/	1
	24	3	97	/	/	1
	120	/	100	/	/	1
	145	/	100	/	/	1
80	24	30	70	/	/	1
	72	/	93	7		1
	168	/	96	1	4	1
	336	/	94	/	6	1
	672	/	15	/	85	1
	1008	/	/	/	100	1

**Figure 1:**
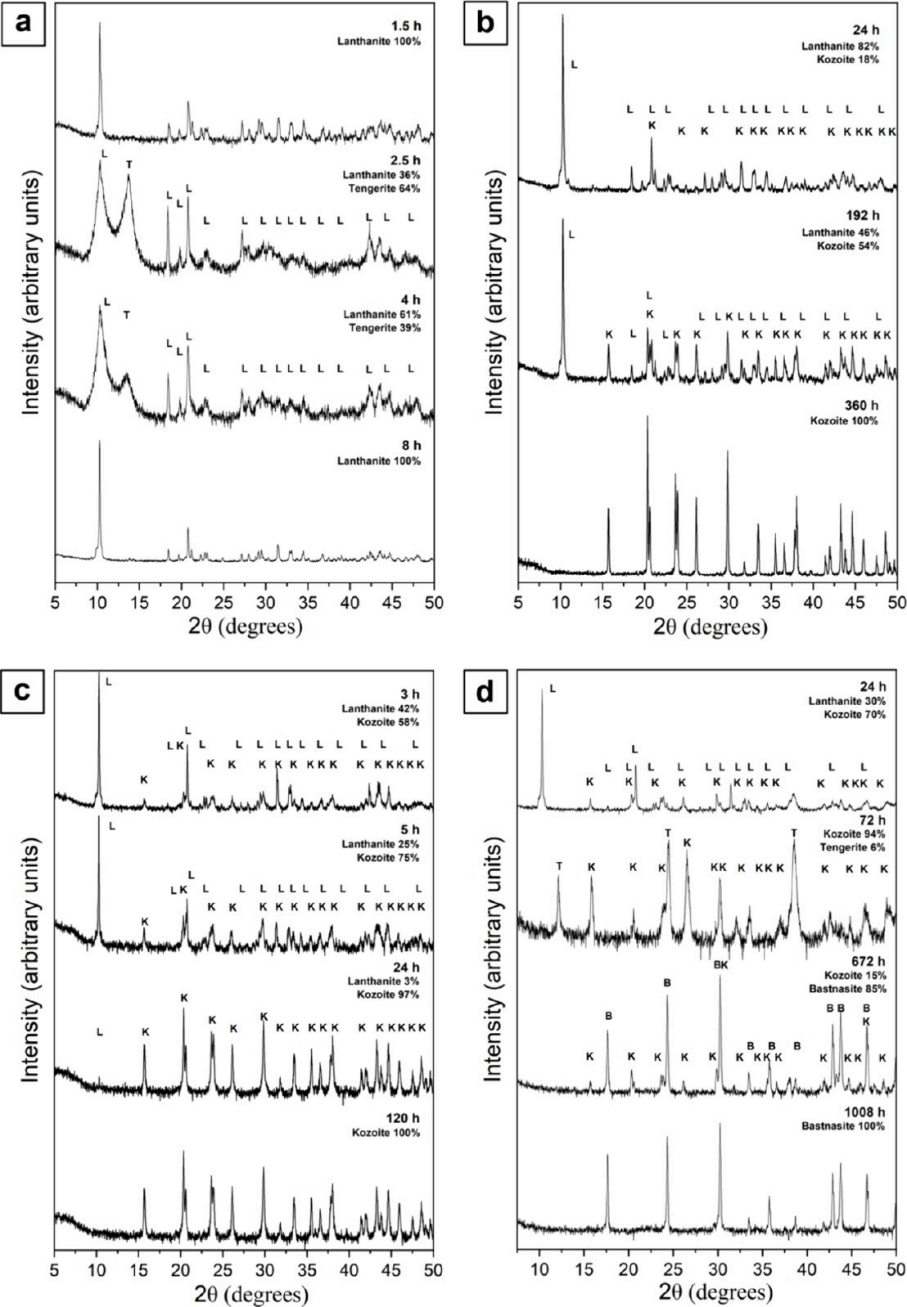
Powder X-ray diffraction patterns showing the transformation of La-carbonates at (a) 21 °C, (b) 35 °C, (c) 50 °C, and (d) 80 °C.

Four crystalline phases were identified in the La system: Lanthanite (La_2_(CO_3_)_3_·8H_2_O; PDF 00–014–0190), tengerite (La_2_(CO_3_)_3_·2–3H_2_O; PDF 00–024–1419), kozoite (LaCO_3_OH; PDF 00–049–0981), and hydroxylbästnasite (LaCO_3_OH; PDF 00–026–0815) (Figure 1). In the lanthanum system, lanthanite was the main crystalline phase at 5 and 21 °C, and an intermediate phase at temperatures ≥35 °C, where it was eventually replaced by more thermodynamically stable phases. Tengerite was present at 21 and 80 °C (Figure 1a,d). Its occurrence at ambient temperature was particularly noteworthy because it formed as a short-lived metastable phase after lanthanite crystallization, coexisting with lanthanite between ∼2.5 and 4 h from the beginning of the experiment and then transforming back to lanthanite (Figure 1a).

The presence of tengerite was primarily indicated by its broad Bragg peak at 12.5° 2θ and was concomitant with the broadening of the 10° 2θ Bragg peak of lanthanite (Figure 1a), a phenomenon not observed in any other crystalline phases of the La system. At 80 °C, tengerite was observed as a metastable secondary phase between 72 and 168 h, coexisting with kozoite and hydroxylbästnasite (Table 1; Figure 1d). Kozoite was found in the experiments at temperatures ≥35 °C. At 35 and 80 °C, it coexisted with lanthanite for up to ∼360 and ∼24 h, respectively, subsequently becoming the only phase present in the system. At 80 °C, kozoite was slowly replaced by hydroxylbästnasite, which became the final phase after 42 days (Figure 1d).

**Figure 2:**
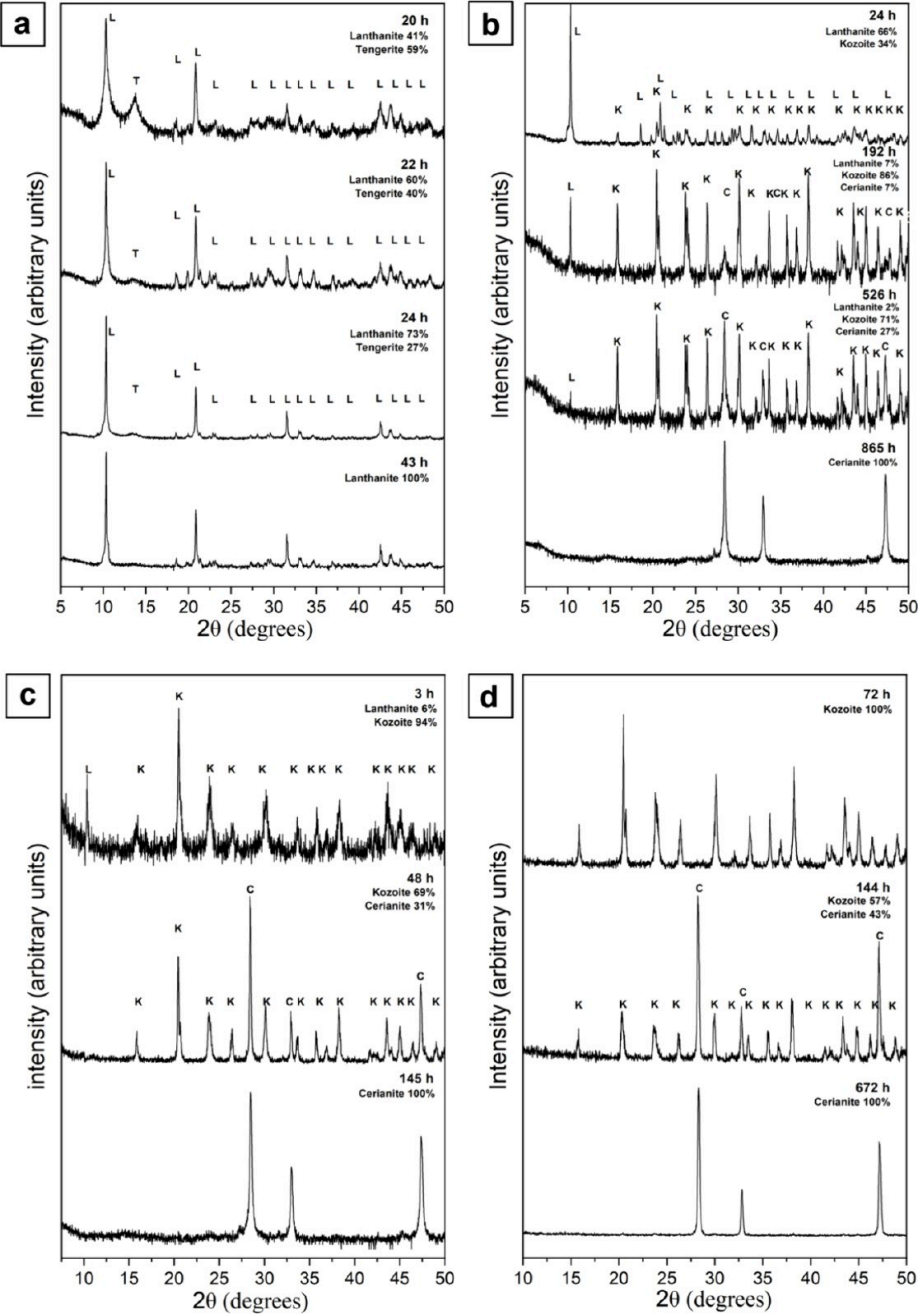
Powder X-ray diffraction patterns showing the transformation of Ce-carbonates at (a) 21 °C, (b) 35 °C, (c) 50 °C and (d) 80 °C.

In the Ce system, the crystalline phases identified were lanthanite, tengerite, kozoite, and cerianite (CeO_2_; PDF 00–043–1002) (Figure 2). Lanthanite was identified as the main crystalline phase at 5 and 21 °C, as an intermediate phase at temperatures ≥35 °C, and even in trace quantities (1%) at 80 °C (Table 2). Tengerite was present only at 21 °C and, similarly to the La system, it coexisted with lanthanite for a short period of time (<20 to ∼24 h). The occurrence of tengerite in the Ce system was also evidenced by its broad peak at 12.5° 2θ and was concomitant with a temporary increase in the broadness of the 10° 2θ peak of lanthanite (Figure 2a). Kozoite was found in the 35, 50, and 80 °C experiments as an intermediate phase sometimes coexisting with lanthanite before transforming into cerianite after >700 h at 35 °C and >144 h at 50 and 80 °C (Figure 2b–d). Cerianite was the most thermodynamically stable phase of the Ce system and it was found in the 35, 50, and 80 °C experiments after long reaction times (Figure 2b–d; Table 2).

**Table 2:** Quantification of Crystalline Phases during the Ce-Carbonate Crystallization Experiments at Different Temperatures

Ce							
*T* (°C)	time (h)	lanthanite wt %	kozoite wt %	tengerite wt %	cerianite wt %	hydroxylbastnäsite wt %	reactor(s)
5	28	100	/	/	/	/	1
	32	100	/	/	/	/	1
	48	100	/	/	/	/	1
	73.5	100	/	/	/	/	1
	147	100	/	/	/	/	1
21	20	41	/	59	/	/	1
	22	60	/	40	/	/	1
	24	73	/	27	/	/	1
	43	100	/	/	/	/	1
	48	100	/	/	/	/	1,2
	64	100	/	/	/	/	1
	90	100	/	/	/	/	1
	144	100	/	/	/	/	1,2
	164	100	/	/	/	/	1
	168	100	/	/	/	/	1
	337	100	/	/	/	/	2
	505	100	/	/	/	/	1,2
	671	100	/	/	/	/	2
	815	100	/	/	/	/	2
35	24	66	34	/	/	/	1
	50	10	90	/	/	/	1
	192	7	86	/	7	/	1
	360	3	81	/	16	/	1
	526	1	71	/	28	/	1
	696	/	20	/	80	/	1
	865	/	/	/	100	/	1
	1201	/	/	/	100	/	1
50	0.75	73	27	/	/	/	1
	3.25	6	94	/	/	/	1
	5.25	/	98	/	2	/	1
	24	/	89	/	11	/	1
	48	/	69	/	31	/	1
	120	/	9	/	91	/	1
	145	/	1	/	99	/	1
80	24	1	99	/	/	/	1
	72	/	100	/	/	/	1
	144	/	57	/	43	/	1
	672	/	/	/	100	/	1

In the Pr system, the only crystalline phases identified were lanthanite and kozoite. The former was the only phase present at both 5 and 21 °C and coexisted with kozoite at higher temperatures (35 and 50 °C) (Table 3). Kozoite instead was the primary phase at 35, 50, and 80 °C (Table 3), eventually becoming the only phase present in the system after long reaction times. No tengerite or hydroxylbastnäsite were detected in this system neither at the highest tested temperature nor after long experimental times.

**Table 3:** Quantification of Crystalline Phases during the Pr-Carbonate Crystallization Experiments at Different Temperatures

Pr						
*T* (°C)	time (h)	lanthanite wt %	kozoite wt %	tengerite wt %	hydroxylastnäsite wt %	reactor(s)
5	29	100	/	/	/	1
	32	100	/	/	/	1
	36	100	/	/	/	1
	52	100	/	/	/	1
	78	100	/	/	/	1
	151	100	/	/	/	1
21	25	100	/	/	/	1
	28	100	/	/	/	1
	32	100	/	/	/	1
	48	100	/	/	/	1
	71	100	/	/	/	1
	144	100	/	/	/	1
	168	100	/	/	/	1
	337	100	/	/	/	1
	505	100	/	/	/	1
	671	100	/	/	/	1
	815	100	/	/	/	1
35	24	47	53	/	/	1
	50	27	73	/	/	1
	192	2	98	/	/	1
	360	1	99	/	/	1
	526	1	99	/	/	1
	670	/	100	/	/	1
	696	/	100	/	/	1
	865	/	100	/	/	1
	1201	/	100	/	/	1
50	1	/	/	/	/	1
	2	79	21	/	/	1
	4	1	99	/	/	1
	6	/	100	/	/	1
	24	/	100	/	/	1
	48	/	100	/	/	1
	120	/	100	/	/	1
	145	/	100	/	/	1
80	144	/	100	/	/	1
	336	/	100	/	/	1
	2520	/	100	/	/	1

In the Nd system, the crystalline phases detected were lanthanite, tengerite, and kozoite. Lanthanite was present only in the 5, 21, and 35 °C experiments (Table 4). In the last one, it is the predominant phase present until time <526 h after which it becomes a secondary phase associated with kozoite and tengerite. Tengerite was detected only at 35 and 50 °C as an intermediate phase. In the first case, it was found after 670 h associated with lanthanite and kozoite while in the second it was the only and major phase at time ≤120 h. In both experiments, it was replaced by kozoite with the ongoing crystallization reaction. Kozoite was present at temperatures ≥35 °C (Table 4). At 35 °C it was found after 670 h and at 50 °C after 120 h becoming the predominant phase in the late stages of the crystallization reactions. At 80 °C it was the only phase detected during all of the duration of the experiment.

**Table 4:** Quantification of Crystalline Phases during the Nd-Carbonate Crystallization Experiments at Different Temperatures

Nd						
*T* (°C)	time (h)	lanthanite wt %	kozoite wt %	tengerite wt %	hydroxylbastnäsite wt %	reactor(s)
5	29	/	/	/	/	1
	32	100	/	/	/	1
	36	100	/	/	/	1
	52	100	/	/	/	1
	77.5	100	/	/	/	1
	151	100	/	/	/	1
21	25	100	/	/	/	1
	28	100	/	/	/	1
	32	100	/	/	/	1
	48	100	/	/	/	1
	70.5	100	/	/	/	1
	144	100	/	/	/	1
	168	100	/	/	/	1
	337	100	/	/	/	1
	505	100	/	/	/	1
	671	100	/	/	/	1
	815	100	/	/	/	1
35	24	100	1	1	/	1
	50	100	1	1	/	1
	192	100	1	1	/	1
	360	100	1	1	/	1
	526	100	1	1	/	1
	670	25	40	35	/	1
	696	/	50	50	/	1
	865	/	80	20	/	1
	1201	/	93	7	/	1
50	1	/	/	/	/	1
	2	/	/	/	/	1
	3.5	/	/	1	/	1
	5.5	/	/	100	/	1
	24	/	/	100	/	1
	48	/	/	100	/	1
	120	/	50	50	/	1
	145	/	80	20	/	1
80	144	/	100	/	/	1
	336	/	100	/	/	1
	2520	/	100	/	/	1

### UV–Vis Experiments

**Figure 3:**
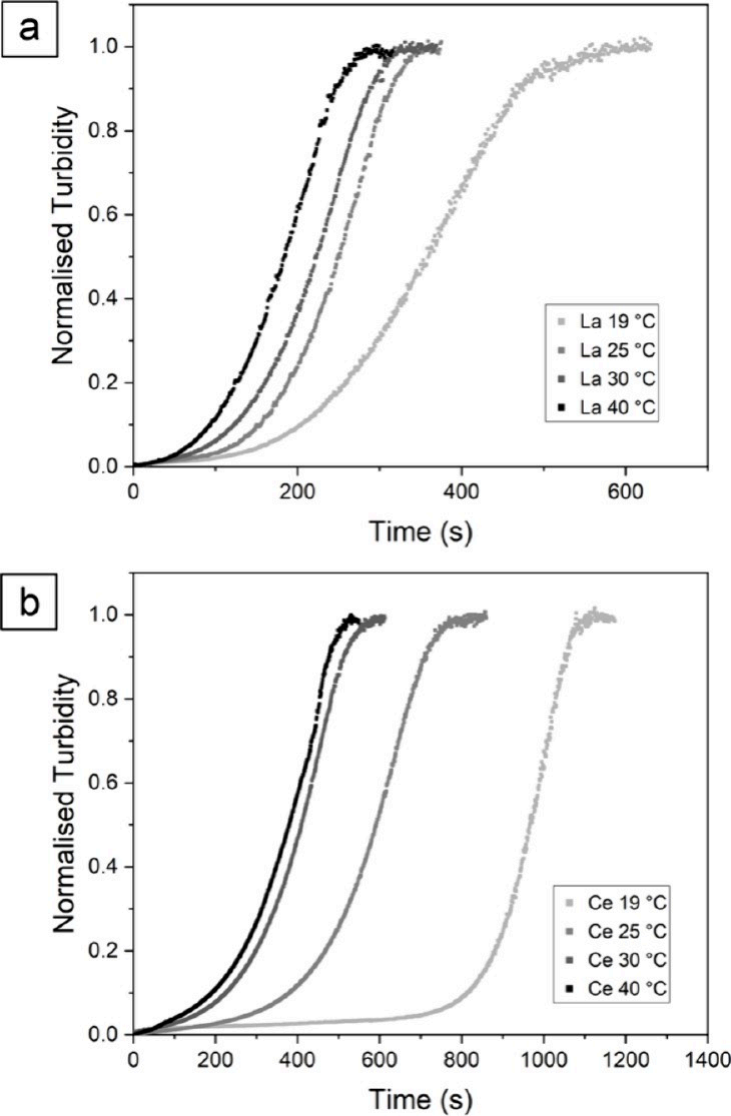
Normalized turbidity graphs showing the crystallization curve for (a) (La)-lanthanite and (b) (Ce)-lanthanite at different temperatures (19–40 °C).

All the UV–vis experiments exhibited a constant general behavior. This was characterized first by an initial sudden increase in turbidity due to the formation of amorphous La or Ce carbonates (ALaC or ACeC) at ∼0–10 s. After that, a more stable sluggish stage takes place where there are no major changes in the level of turbidity. This phase spans from *t* ∼ 10–110 s at 19 °C and from *t* ∼ 10–30 s at 40 °C for the La system and from *t* ∼ 10–650 s at 19 °C and from *t* ∼ 10–70 s at 40 °C for the Ce system (Figure SI-1). Subsequently, the turbidity started to increase again until ultimately reached a maximum (Figure 3).

The study of (La)- and (Ce)-lanthanite crystallization kinetics at five different temperatures (19, 25, 30, and 40 °C) revealed rates of crystallization that increased proportionally with rising temperatures, with the La system exhibiting the fastest reaction times and followed by the Ce system with slightly longer ones. The slow initial stage of the reactions, characterized by the presence of the amorphous precursors prior to their transformation to crystalline (La)- and (Ce)-lanthanite, was always shorter for the La system compared to Ce at each tested temperature (see Table 5). Similarly, the end of the primary crystallization reaction (maximum absorbance) in the La system was reached at approximately 630 s at 19 °C and 290 s at 40 °C. In the Ce system, this time range spanned from approximately 1130 s at 19 °C to around 530 s at 40 °C.

**Table 5:** Values of Induction (*t*_0_) and Ending Time (*t*_end_), Reaction Rate (*k*), and Activation Energies of Crystallization (*E*_a(cryst)_) and Nucleation (*E*_a(nucl)_) Obtained from the REE-Carbonates Crystallization Reactions^a^

	*T* (°C)	*t*_0_(s)	*t*_end_(s)	*k* (×10^–3^s^–1^)	*E*_a(cryst.)_kJ/mol	*E*_a(nucl.)_kJ/mol
La	**19**	109	629	2.531	21(5)	37(3)
	**25**	92	359	3.630		
	**30**	55	328	4.059		
	**40**	31	293	4.722		
Ce	**19**	654	1124	0.998	32(9)	40(2)
	**25**	255	806	1.470		
	**30**	133	588	2.176		
	**40**	76	525	2.367		
Pr	**19**	1600	4860	0.331	18(16)	48(8)
	25 (*)	1200	3820	0.286		
	30 (*)	950	2505	0.298		
	35 (*)	550	2575	0.506		
Nd	**19**	12,800	53820	0.039	27(13)	42(5)
	25 (**)	10,000	48850	0.034		
	45 (**)	4200	26270	0.047		
	55 (**)	1800	7980	0.155		

a *t*_end_ refers to the moment when the maximum value of absorbance was reached during the crystallization reactions. (*) Minor amounts (∼1 wt %) of kozoite and (**) tengerite detected at the end of the experiment.

Unlike the La and Ce systems, XRD analysis of the resulting solids from the Pr and Nd experiments revealed the presence of small amounts (∼1 wt %) of kozoite and tengerite in addition to lanthanite at temperatures ≥25 °C. Therefore, UV–vis monitoring of (Pr)- and (Nd)-lanthanite formation in situ and in real-time was affected by the formation of these two phases. However, the comparison of the UV–vis turbidity curves of all lanthanites at 19 °C showed that the kinetics of lanthanite crystallization was directly proportional to temperature and inversely proportional to the atomic mass of the specific REE used (Figure SI-2; Table 5).

### Synchrotron-Based Experiment

**Figure 4:**
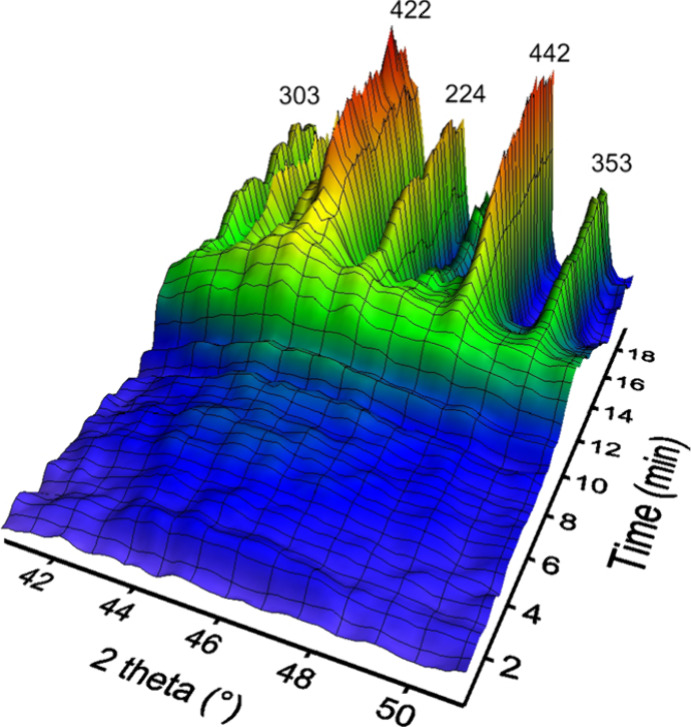
3D plot of a selected time-resolved WAXS pattern showing the crystallization of (Ce)-lanthanite.

**Figure 5:**
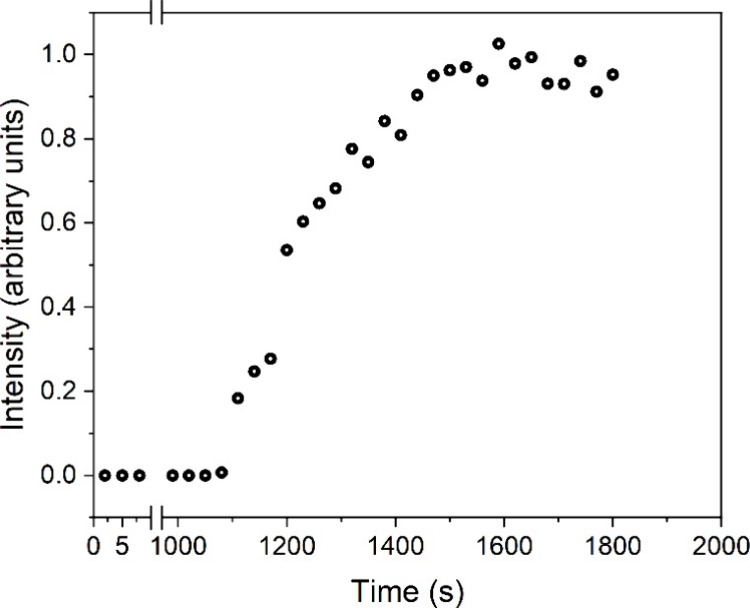
(Ce)-lanthanite crystallization curve derived from the analysis of the WAXS data.

The WAXS data allowed for in situ, time-resolved monitoring of (Ce)-lanthanite crystallization from solution via an amorphous Ce-carbonate (ACeC) intermediate. The data revealed the growth of Bragg peaks corresponding to (Ce)-lanthanite, with peak intensity reaching its maximum ∼18 min after the experiment began, indicating the completion of the primary crystallization reaction. No other crystalline phases were detected. Figure 4 shows a 3D plot of a selected time-resolved WAXS pattern. At the onset of crystallization, between 12 and 14 min after the beginning of the experiment, a small increase in intensity was observed. This transient broad hump in the WAXS pattern was interpreted as the combined scattering from lanthanite nanoparticles, which were too small to generate sharp and distinct Bragg peaks. The (Ce)-lanthanite crystallization curve derived from the analysis of the WAXS data was very similar to the one obtained using UV–vis spectrophotometry (Figure 5).

### FTIR Analysis

**Figure 6:**
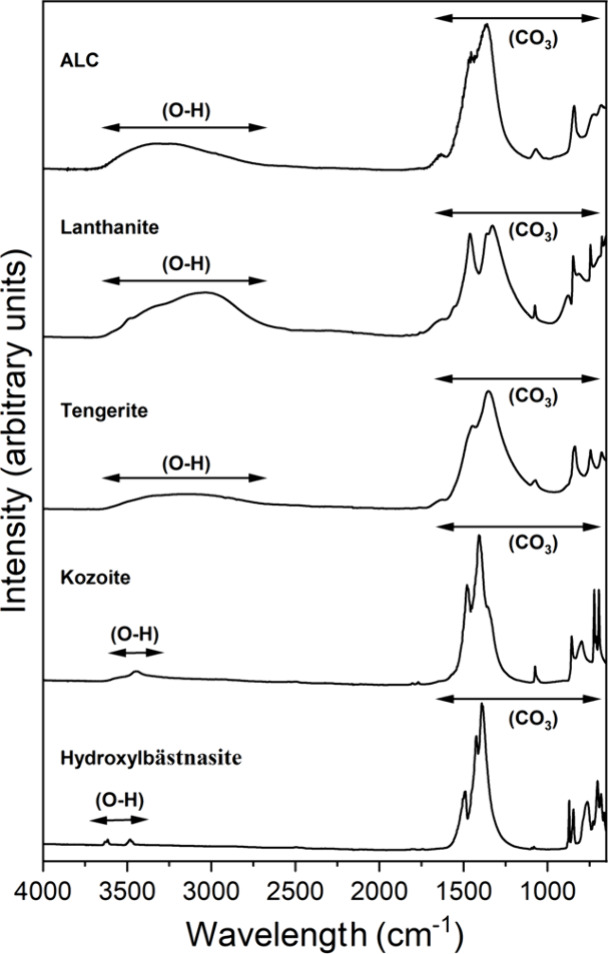
FTIR spectra of amorphous La carbonate (ALaC), (La)-lanthanite, (La)-tengerite, (La)-kozoite, and (La)-hydroxylbastnäsite showing the position of the OH bands and of the main carbonate bands.

The FTIR analyses of the solid samples contributed to their identification and revealed structural differences (Figure 6). The IR bands in the region comprised between ∼1500 and 700 cm^–1^ are mostly corresponding to the main stretching vibrations of the carbonate ions.^12,13,74^ The other dominant feature is the bands located between ∼2500 and 3700 cm^–1^, which correspond to the O–H group of structural water in the solids.^12,12,74^ This region of the FTIR spectrum shows the most significant variations among the identified phases. The broad band located between ∼2500 and 3700 cm^–1^ narrows and decreases in area in the order: lanthanite > amorphous carbonate > tengerite. Also, the presence of the O–H group is indicated by a narrower peak at ∼3440 cm^–1^ for kozoite and by two small bands (3500 and 3600 cm^–1^) for hydroxylbastnäsite.

### SEM Analysis

**Figure 7:**
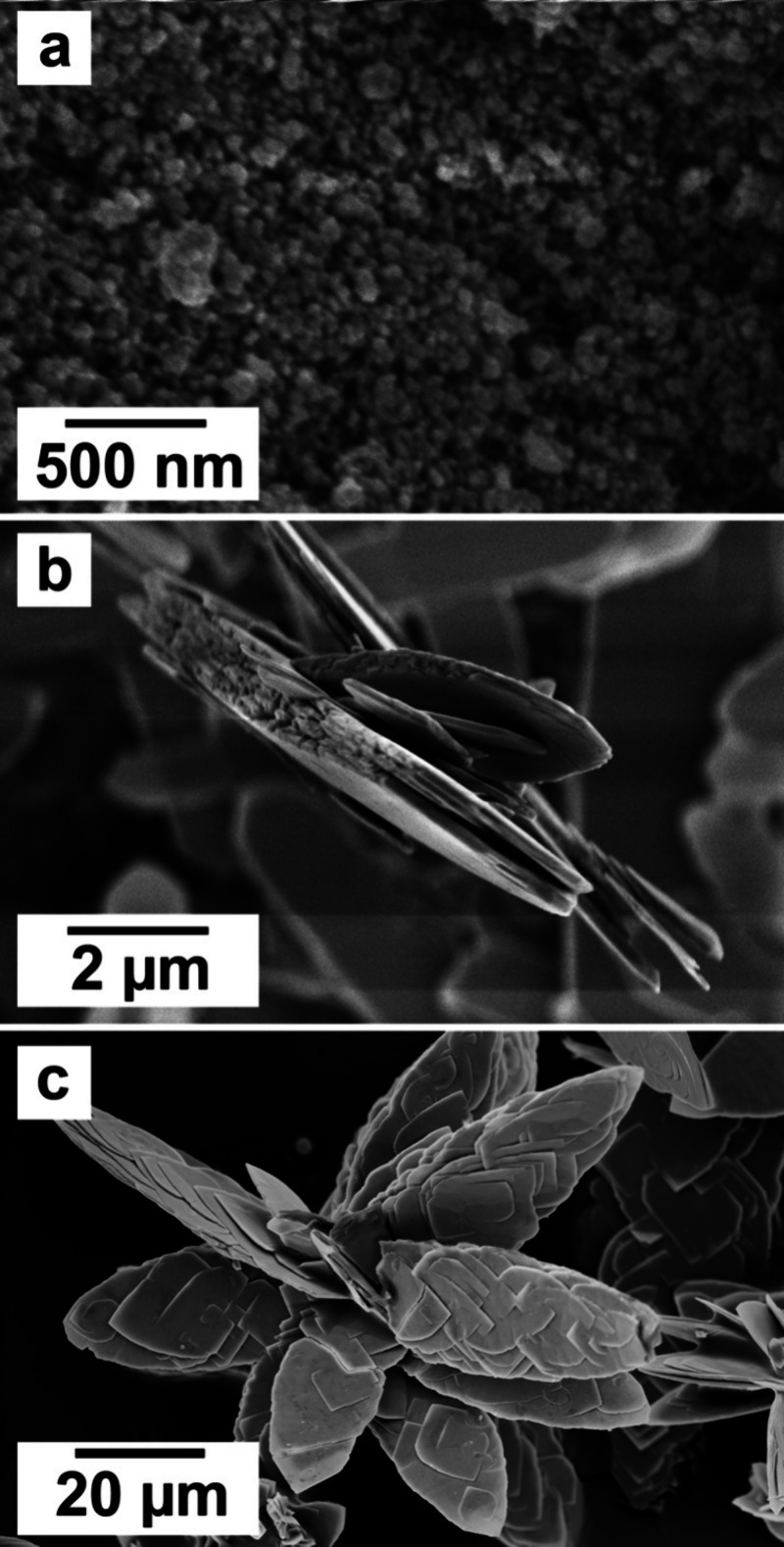
Crystallization of (Ce)-lanthanite from solution from (a) a poorly ordered carbonate precursor (after 0.5 h), resulting in lanthanite aggregates (after 2 h) (b) that increase in size and develop platy morphologies (c) (after 24 h). All the images refer to the experiment carried out at ambient temperature (21 °C).

**Figure 8:**
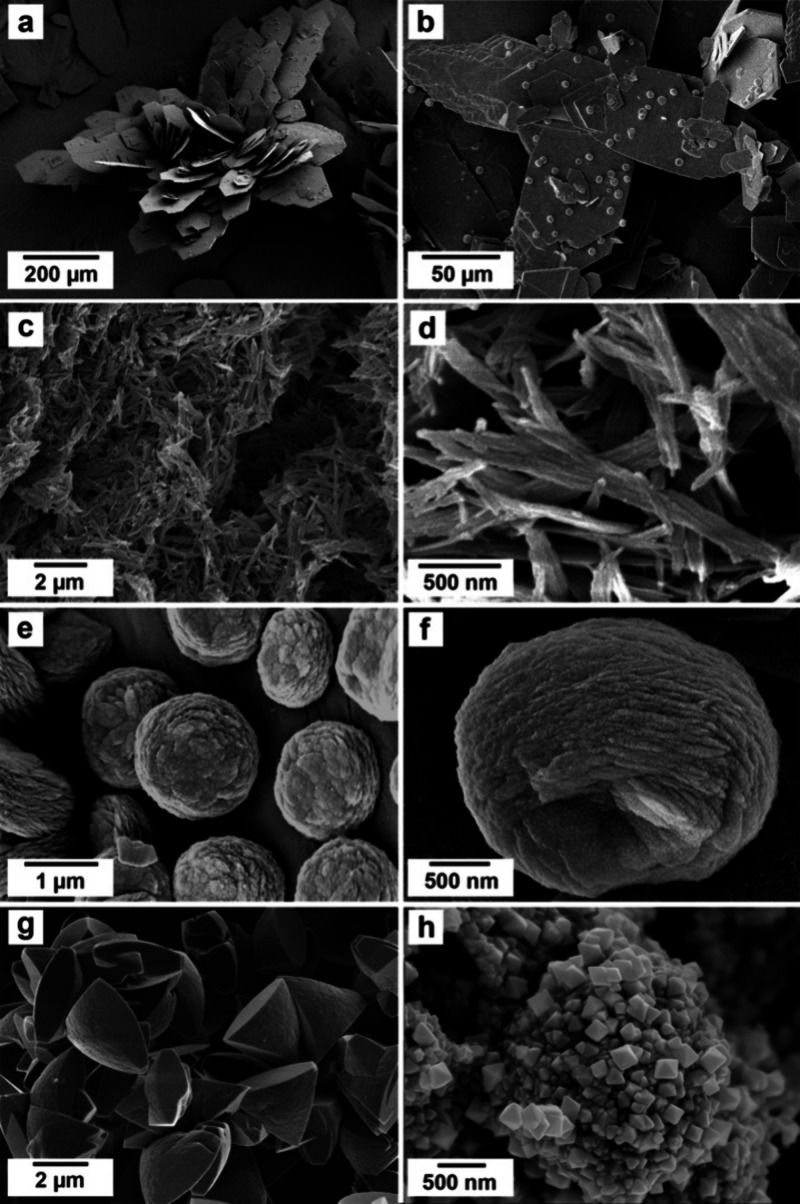
SEM images of REE-bearing carbonates formed during crystallization from solution experiments. (a) (La)-lanthanite at ambient temperature after 815 h; (b) spherulitic crystals of (La)-kozoite on the surface of (La)-lanthanite at 35 °C after 24 h; (c, d) (Nd)-tengerite obtained at 50 °C after 48 h; (e, f) details of sperulitic kozoite on the surface of lanthanite at 35 °C after 50 h; (g) (La)-hydroxylbastnäsite crystals (triangles) at 80 °C after 1008 h; (h) cerianite nanocrystals at 35 °C after 696 h.

**Figure 9:**
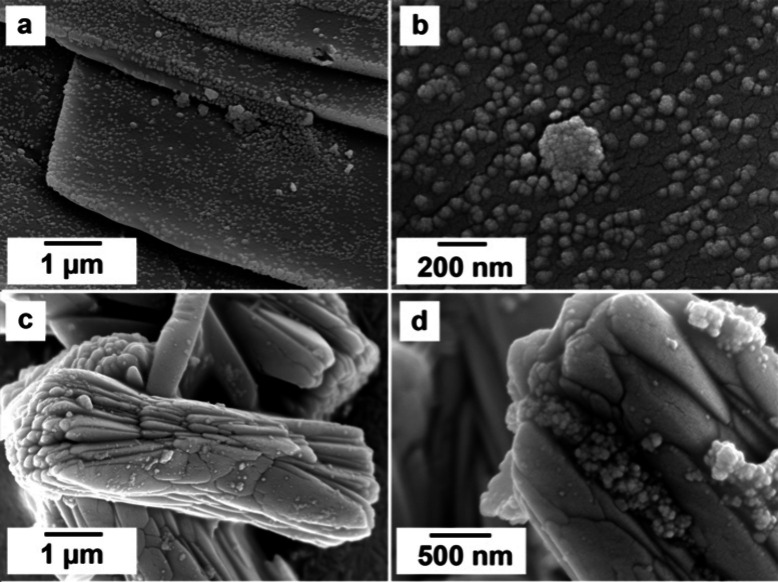
(a, b) Nanocerianite crystals growing on the surface of (Ce)-lanthanite at 35 °C after 192 h; (c, d) bow tie shaped (Ce)-kozoite with nanocerianite crystals growing on its surface at 35 °C after 360 h.

The SEM analysis carried out on the final products of the homogeneous mixing experiments allowed one to study and evaluate the morphology of the different crystalline phases present and their changes at different temperatures and reaction stages (Figures 7 and 8). High-resolution images showed that the all-amorphous REE-carbonate precursor, present at the onset of the crystallization reactions, consisted of tiny spheres of <50 nm in size (Figure 7a). (La)-, (Ce)-, and (Pr)- lanthanites developed a platy morphology with dimensions between 10 and 50 μm (Figures 7b,c and 8a).

(Nd)-tengerite obtained at 35 and 50 °C consisted of acicular nanoaggregates with a length <2 μm and thicknesses of <200 nm, containing subunits of 100–200 nm in length (Figure 8c,d). Generally speaking, (La)-, (Ce)-, (Pr)-, and (Nd)-kozoite showed temperature-dependent morphologies. At ambient or low temperatures, the crystals were generally attached to the lanthanite surfaces (Figure 8a,b) and showed spherulitic morphologies, consisting of 1–2 μm nanoaggregates made of crystals with sizes >100 nm (Figure 8e,f). At higher temperatures, kozoite consisted of bow tie crystalline nanoaggregates (Figure 9c,d) with dimensions of 1–3 μm and formed by small elongated nanocrystals of <800 nm in length. (La)-hydroxylbastnäsite and cerianite did not show any major morphological variability: (La)-hydroxylbastnäsite exhibited triangular shapes with sizes of 2–3 μm (Figure 8g) whereas cerianite consisted of octahedral prisms with dimensions ranging between 50–200 nm, sometimes forming spherulitic nanoaggregates (Figure 8h) and always growing on the surface of lanthanite (Figure 9a,b) and kozoite (Figure 9c,d).

### Reversible Lanthanite-Tengerite Crystallization

**Figure 10:**
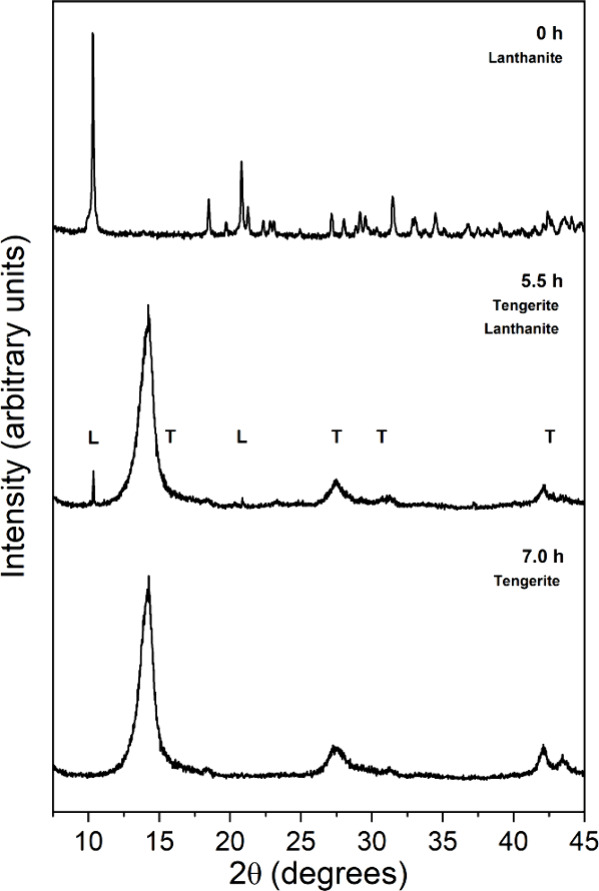
XRD patterns showing the progressive dehydration of (La)-lanthanite completed in a period of 7 h at 80 °C.

**Figure 11:**
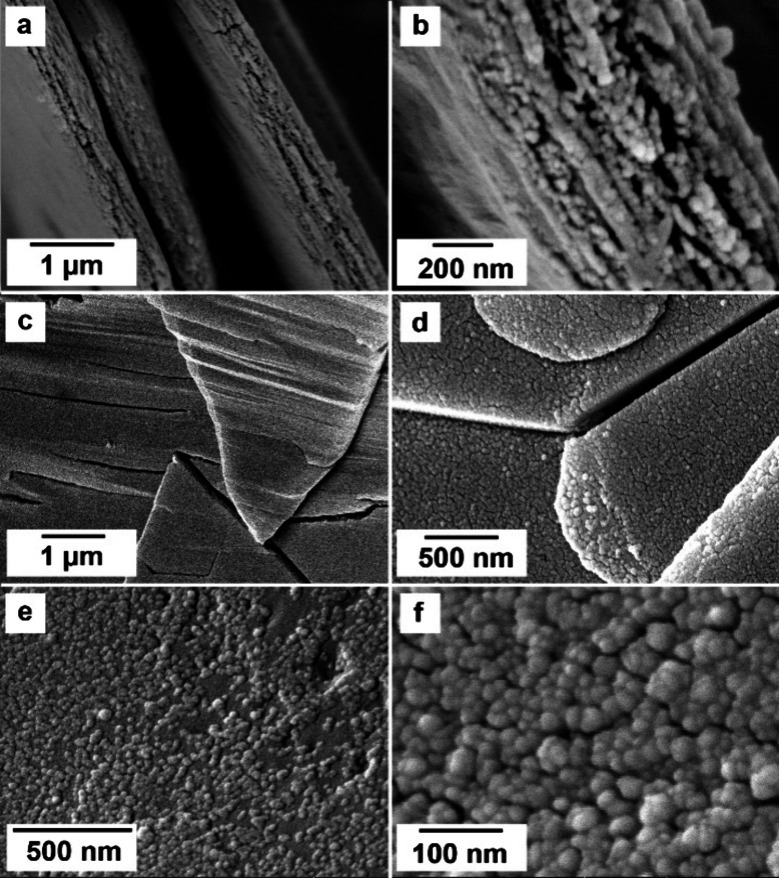
Dehydration of (La)-lanthanite forming nanoparticles of (La)-tengerite. (a, b) Breakdown to (La)-tengerite nanocrystals along the (La)-lanthanite (001) plane in the aqueous solution experiments after 5.5 h. Images (c) to (f) show the formation of fractures on the surface of lanthanite and the development of nanoparticle domains average size of 27 ± 5 nm in diameter in the solid-state transformation experiments (respectively (c), (d) after 5.5 and (e), (f) after 7 h).

The results of the (La)-lanthanite dehydration experiments carried out at 80 °C revealed a complete solid-state transformation of this mineral into (La)-tengerite after a period of 7 h (Figure 10). This transformation was observed using conventional XRD analyses, whereas SEM observations on the resulting crystals showed some morphological features consistent with dehydration processes. While the overall platy morphology of the initial lanthanite remained largely unchanged, nanoscale cracks with widths ranging from 10 to 100 nm were observed in the final product. Additionally, a substructure composed of nanoparticles, averaging 27 ± 5 nm in diameter (*N* = 100), was observed (Figure 11).

**Figure 12:**
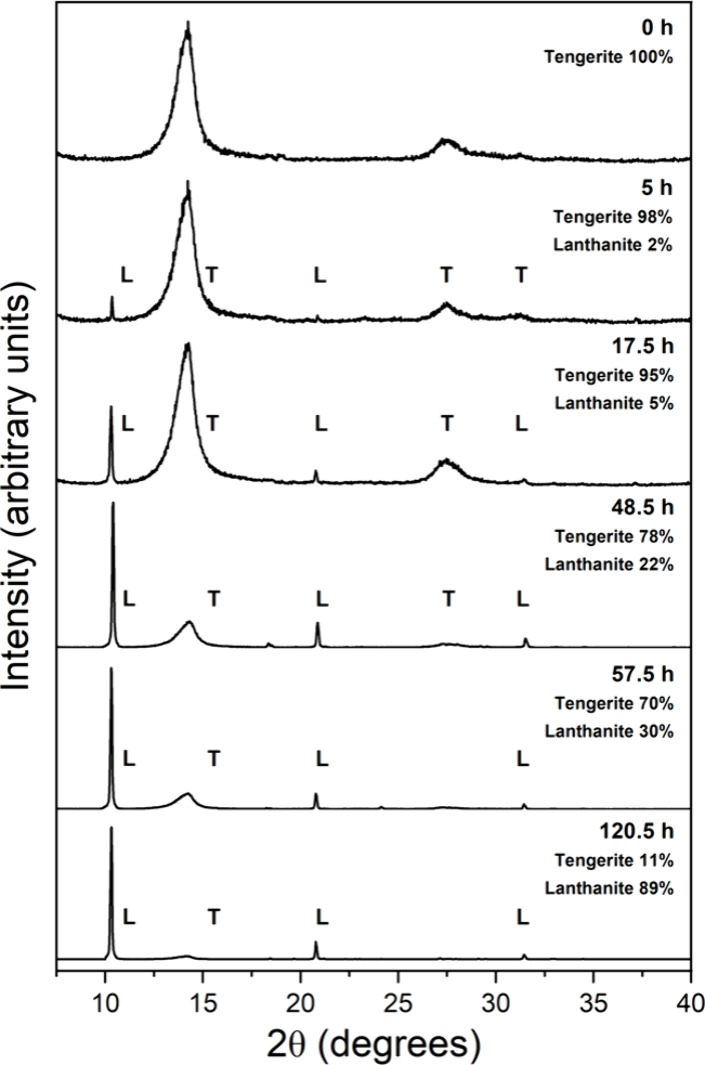
XRD patterns showing the progressive rehydration of (La)-tengerite and transformation to (La)-lanthanite during a period >120 h at 21 °C.

**Figure 13:**
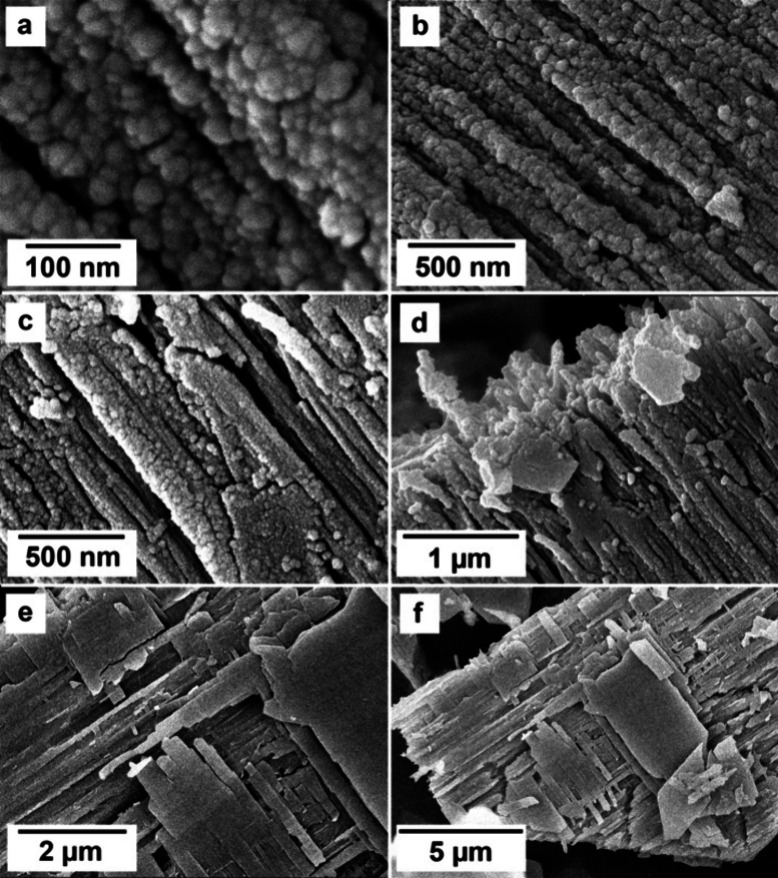
SEM images of rehydration of (La)-lanthanite from (La)-tengerite showing (a, b) an increase in the size of nanoparticles (after respectively 5 and 120 h) and the formation of nano- and microrods (48.5 h) (c, d) transforming into sheets of (La)-lanthanite (e, f) after 120 h.

When tengerite was placed in a reactor containing Milli-Q water at ambient temperature, it transformed back to (La)-lanthanite after a period of more than 120 h, as confirmed by XRD (Figure 12). SEM observations revealed the (La)-tengerite to (La)-lanthanite transformation through the progressive development of new features: first, an increase of the size of the nanoparticles from 27 ± 5 to 41 ± 7 nm after 120 h. Second, the growth of these nanoparticles into rods, needles, and plates (Figure 13).

## Discussion

Our data suggest that the formation of REE-carbonates toward the hydroxylbastnäsite has greater complexity than previously assumed. The early stages of REE-carbonate crystallization involve multiple crystalline phases and reversible reactions. These transformations are mediated by aqueous solution, highlighting the intricate interplay among temperature, solution chemistry, and phase stability. The different ionic radius of the REE is a crucial factor in controlling crystallization pathways, metastable phases, and the thermodynamically stable end phase at different temperatures.

### Lanthanite Crystallization from Amorphous Precursors

**Figure 14:**
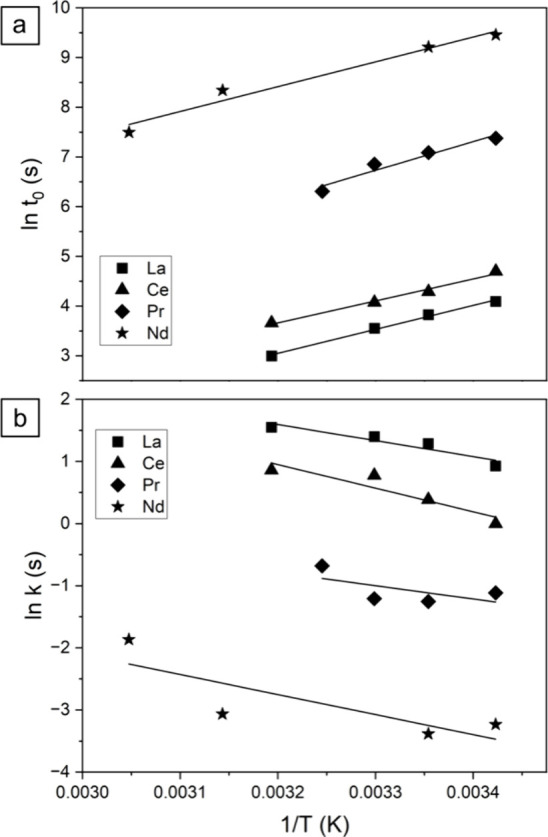
Arrhenius plots for lanthanite nucleation (above) and crystallization (below).

The patterns of the turbidity profiles showed a general sigmoidal trend in all the experiments and at the different temperatures tested (19–55 °C). These fit properly in the JMAK model (*R*^2^ > 94%) using one value of *n* and *k* for the main crystallization mechanism. When the data is plotted as a function of −ln ln(1 – α) against ln *t*, it gives close to parallel lines (Figures SI-3–SI-6). In our experiments, the lines resulted to be close to parallel for La and Ce (slope difference <10%) and almost close to parallel for Pr and Nd (respectively slope difference 12 and 10%). These moderate variations in the slope can be explained by the fact that in our Pr and Nd experiments, the reaction curves were influenced by the crystallization of minor amounts of (Pr)-kozoite and (Nd)-tengerite above 19 °C at the end of the primary crystallization process. Furthermore, even if properly carried out, generally UV–vis experiments are not completely free from slight instrumental errors which can be a consequence of several factors (e.g., minor fluctuation of the ambient temperature, false reading of the instrument because of the presence of slightly larger crystals or polycrystalline aggregates in the reactor, etc.). The sum of the “errors” given by the presence of minor/trace amounts of the secondary phases present in the final product of Pr and Nd experiments and the minor “instrumental errors” can give an explanation about why the plots seem to be less parallel compared to the La and Ce ones. All things considered and net of the errors mentioned above, our results demonstrate that the crystallization mechanism is the same regardless of the temperature and the REE composition of the lanthanite.^60,61^ The calculation of the activation energies of nucleation and crystallization using an Arrhenius approach (Figure 14; Table 5), gives values for the lanthanite energy of nucleation between 37 and 48 kJ/mol, while the energy of crystallization between 21 kJ/mol (La) and 32 kJ/mol (Ce).

Although the calculated values are very similar for all tested REEs (Table 5), particularly for the activation energy of nucleation, the activation energies for (Pr)- and (Nd)-lanthanites may be influenced by the crystallization of small amounts of (Pr)-kozoite and (Nd)-tengerite above 19 °C at the end of the primary crystallization process. Therefore, the values for these two heavier REEs should be interpreted with caution. However, the UV–vis data clearly showed a general trend in which the induction times and rates of crystallization were directly proportional to the ionic radii of the REE and inversely proportional to the temperature for each single REE (Table 5; Figure 3).

This behavior can be explained by the specific ionic potentials of the trivalent REE ions, which can be calculated by dividing the ion’s valence by its ionic radius. Due to the phenomenon known as lanthanide contraction,^2^ REE^3+^ ionic radii decrease moving through the lanthanide series from La to Lu (atomic numbers 57–71). This is then translated into an increment in the ionic potential with the increase in the REE atomic number. While in solution, all ions are solvated, but prior to the initiation of the nucleation of a crystal or the incorporation in a crystalline structure, an ion needs to lose its hydration shell. The ionic potential of the REE^3+^ cation determines its ability to retain water molecules in its solvation shells.^14,75,76^ The higher the ion’s ionic potential, the higher the energy required to dehydrate it before incorporating it into a mineral structure. Thus, an ion with a higher ionic potential requires higher temperatures or longer times for the nucleation and completion of its crystallization reaction.^14,77^ The ionic potentials calculated for La^3+^, Pr^3+^, Ce^3+^, and Nd^3+^ are 2.60, 2.70, 2.74, and 2.77 Å^–1^ respectively.^78^ La^3+^ would require the least energy for dehydration, followed by Ce^3+^, Pr^3+^, and Nd^3+^. For this reason, the beginning of crystallization for a lighter REE-carbonate takes less time compared to the heavier ones. Evidence of this fact can also be found in the lifetime of the amorphous REE-carbonate precursors. Our UV–vis data, accordingly with results reported by the study of Vallina et al.,^13^ show that at 19 °C there are two orders of magnitude of difference between the lifetime of ALaC and ANdC (respectively ∼9 and ∼900 min). It is also known that amorphous REE-carbonate precursors involving Dy^3+^ and Yb^3+^ (ionic potentials 3.03 Å^–1^ for Dy^3+^ and 3.22 Å^–1^ for Yb^3+^), show longer lifetimes of ∼3000 min at ambient temperature for amorphous Dy carbonate^12^ and >10,000 min at 120 °C for amorphous Yb carbonate.^79^ The lifetimes clearly increase proportionally with the ionic potential of the REE^3+^ ions, with ALaC requiring less energy for dehydration compared to other amorphous precursors containing heavier REE^3+^ ions with larger ionic potentials. A comparable behavior is known to happen between Ca^2+^ and Mg^2+^ amorphous carbonates, with the former being stable for <2 min^23,24^ and the Mg-bearing amorphous calcium carbonate for several hours or days^80,81^ at ambient temperature. Similarly to the REE^3+^ ions, the higher stability of the Mg-bearing amorphous calcium carbonate was attributed to its larger ionic potential (3.07 Å^–1^^82^) compared to Ca^2+^ (2.02 Å^–1^^23^) and its stronger hydration shell^83^ requiring higher energy to dehydrate.

### Role of Structural Water in the Lanthanite-Tengerite Reversible Transformation

The crystallization from solution experiments, including the synchrotron-based experiment showed that lanthanite was the predominant phase at lower temperatures (5 and 21 °C) after the breakdown of the amorphous precursor. However, lanthanite undergoes a reversible dehydration process and a partial transformation to tengerite in solution, evident at both the atomic and nanoscale levels.

Although the structure of tengerite has not been well described, both lanthanite and tengerite are considered related to each other because they are both orthorhombic minerals with very similar chemistry, layered structures, and carbonate ions coordinated to REE ions. Their coordination environment and the number of H_2_O molecules differ, but both minerals share the presence of weak hydrogen bonds and potentially disordered states of some of their H_2_O molecules.

Lanthanite structure (*Pccn* space group) consists of layers of RE-O coordination polyhedra and carbonate groups,^27,33,35^ which are parallel to the (010) plane and connected to one another by hydrogen bonds. This arrangement contributes to its strong {010} cleavage.^27^ This mineral has 4 structurally independent water molecules, three of them forming relatively strong hydrogen bonds within the layers and between adjacent layers. However, the fourth water molecule W(4) is notable for its distance of 2.891 Å to another W(4) site, indicating a potential disordered bond between them that may favor dehydration.^27,33^

In comparison, the tengerite structure (*Bb*21*m* space group) has layers forming a more integrated three-dimensional framework through additional carbonate linkages.^84,85^ In this mineral, one H_2_O molecule is directly coordinated to the REE atom as part of its 9-fold coordination with the carbonate groups. This coordination involves the water molecule participating in hydrogen bonding, which influences the overall stability and structure of the mineral. Miyawaki et al.^85^ suggested that certain water molecules are loosely held within the mineral structure and can be gained or lost without significant structural alteration, similar to zeolitic water. This would explain its variability in water content from 2 to 3 molecules.

We suggest that the reversible transformation between lanthanite and tengerite in our ambient temperature experiments is a consequence of a dynamic interplay between thermodynamic stability and kinetic factors. Thermodynamic stability determines which mineral is most stable under given conditions (e.g., pressure, temperature), while kinetic factors, such as dehydration rates and surface energy minimization, influence the actual formation and transformation rates. The final crystal structure is thus determined by the balance between these thermodynamic and kinetic influences. We can outline three main steps that explain the entire process:

1. Primary lanthanite crystallization: The initial formation of an amorphous phase upon mixing solutions is common in crystallization processes and has been followed not only in REE-carbonates^8,12,13^ but also in many other Ca–Mg carbonates (e.g., refs (23,80,86)). This amorphous REE-bearing carbonate phase forms at high supersaturation conditions and serves as a precursor for the subsequent crystallization of lanthanite, as evidenced by synchrotron-based XRD experiments (Figure 4, Figure 5). Our data show no other crystalline phase forming before primary lanthanite.
2. Partial dehydration of lanthanite: The XRD data indicated a transformation from lanthanite (La_2_(CO_3_)_3_·8H_2_O) to tengerite (La_2_(CO_3_)_3_·2–3H_2_O), a relatively fast structural change that was triggered by a dehydration process happening within a few hours from the beginning of the crystallization experiment (<2.5 h for La system and <20 h for Ce system; respectively see images 1a and 2a). The imperfections in the (010) planes of lanthanite can facilitate dehydration at these sites with minimal energy variation. This dehydration-driven transformation to tengerite would break down the larger primary lanthanite crystals showing a crystallite size of ∼880 Å into smaller tengerite nanocrystals with a crystallite size of ∼90 Å at the end of the reaction (Table SI-1), leading to Bragg peak broadening of both phases (Figures 1a, and 2a). Solid-state lanthanite-tengerite transformation experiments at 80 °C also support a transformation process involving the loss of structural water molecules and a rearrangement of the crystal lattice.
3. Rehydration of tengerite: The reverse reaction is slower compared to the initial dehydration, taking a few days to complete. This transition from tengerite back to lanthanite implies that tengerite nanocrystals are metastable under experimental conditions and less stable than lanthanite over the long term. The reversibility of this dehydration–rehydration reaction depends on environmental conditions and the energy barriers involved in this process. Within the scope of this work, for “environmental conditions” we mean the experimental conditions created by the interplay of many chemico-physical parameters including temperature, pressure, and the presence of other chemical species in the solution, such as ions or solvents. In the same way, we refer to a “change in the environmental conditions” when there is a substantial external disturbance leading to an alteration in one or more parameters which can influence the dehydration–rehydration process. In our experiments, the parameters are kept constant to ensure that the observed reactions are solely driven by the inherent properties of the materials. In this way, we hypothesize that the activation energies for both the dehydration and rehydration reactions are similar, allowing the system to remain close to equilibrium.

If the activation energies for the forward and reverse reactions are similar and the overall reaction is close to equilibrium, then the dehydration–rehydration process can occur reversibly. Our results show that at 21 °C lanthanite is more thermodynamically stable under the given experimental conditions, as evidenced by the final transformation of all tengerite back to lanthanite. Apart from our experiment result, lanthanite can be considered more stable than tengerite because it is way more commonly found both in nature and in experimental crystallization studies.^8,13−16,30−32^ Furthermore, the specific experiment carried out for studying the lanthanite-tengerite reversible transformation was performed using La as an REE representative. All previous studies showed the absence of tengerite in the La system and that lanthanite is favored when the radius of REE is equal to or larger than Nd (i.e., La, Ce, Pr).^8,13,31,34^ Conversely, tengerite-type carbonate structures are thought to be more typical for heavier REE with ionic radii smaller than Nd (e.g., refs (13,79,84)). This is additional evidence that can support the hypothesis that lanthanite should be more stable than tengerite under our experimental conditions. Although specific surface energy values for these two phases are unknown, this behavior suggests that nanometer-sized tengerite may have higher surface energy, making it less thermodynamically stable over time. The system would minimize its overall energy by transitioning back to lanthanite, which has a larger size and lower surface energy. At the beginning of the rehydration process, the crystallite size of tengerite was ∼90 Å increasing up to ∼100 Å after 120 h, while lanthanite after 120 h showed a crystallite size of 1040 Å (Table SI-1).

Overall, this reversible reaction can be written as
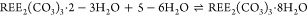


The reversibility and extent of this reaction would also be affected by factors such as the temperature, pressure, and presence of other species in the solution. The thermodynamic data about tengerite in the literature is scarce. In fact, the existing data from Wakita and Nagashima^87^ may not be fully reliable due to discrepancies in crystallographic data.^9^ However, tengerite formation seems to be thermodynamically favored at elevated temperatures (e.g., it forms directly from a poorly ordered precursor at temperatures between 60 and 95 °C, while lanthanite is absent)^13^ and in some of our experiments (e.g., Nd at 35–50 °C) it did not revert to lanthanite because temperature inhibited its rehydration, transforming afterward into the anhydrous phase kozoite.

### Metastable Nanophases Crystallization via Spherulitic Growth

The crystallization from solution experiments demonstrated that kozoite is one of the predominant phases at low hydrothermal temperatures (35 to 80 °C) where it can be associated with other REE-carbonate phases. Its crystalline morphologies were temperature dependent: at ambient and low temperatures, it grew on the lanthanite surfaces and showed typical spherulitic morphologies, whereas, at higher temperatures, it generally showed bow tie morphologies consisting of nanocrystalline aggregates. These morphologies were the result of the rapid spherulitic growth process which is considered to develop through a “secondary nucleation”, usually termed “growth front nucleation”.^88,89^ After the formation of a single nucleus (core) the growth proceeds via the continuous nucleation of new nanoparticles with random orientation on the surface of the growing spherulite.^13,25,88,89^ The result of the process is the crystallization of μm-sized aggregates of nanoparticles with spheroidal morphologies with no structural continuity between the newly nucleated particles and the already-existing ones.^25,88^ Besides REE-carbonates, other carbonate minerals are known to grow via the same mechanism such as vaterite,^24,90^ calcite,^81,91^ aragonite,^92^ monohydrocalcite,^80^ and dolomite.^86^ A high crystallization driving force is required for the spherulitic growth to happen^25,90^ which in aqueous systems, is usually given by high supersaturation levels with respect to the solid phase.^13^ Andreassen et al.^90^ and Beck and Andreassen^91^ suggested that, for carbonate minerals, the saturation index (SI) required to promote the spherulitic growth process should be larger than 2–3. In order to determine saturation indices of the REE-carbonates, the solubility products of the solid phases involved in the reactions are required. However, information about the REE-carbonates solubility products is very limited, only restricted to pure (La)-lanthanite, (Ce)-lanthanite, (Nd)-lanthanite^72^ and (Nd)-kozoite, (La)-hydroxylbastnäsite and (Nd)-hydroxylbastnäsite.^73^ Besides, it is known that the solubility of nm-sized phases can significantly differ from that of bulk crystals (e.g., ref (93)). For this reason and because of the lack of solubility product data for (La)-kozoite in the literature, values for the analogues (Nd)-lanthanite and (Nd)-kozoite were used in our PHREEQC calculations. These showed that the aqueous solution is already supersaturated for kozoite (SI_Nd-koz_ = 2.34) when in equilibrium with (Nd)-lanthanite, a value that is high enough to promote spherulitic growth^90^ and could be maintained if lanthanite dissolves during the transformation process. Therefore, we suggest that the lanthanite-kozoite transformation would happen via a dissolution-recrystallization process coupled with spherulitic growth. This is a surface-driven process that does not involve a significant internal restructuring of the original lanthanite crystals.

Hydroxylbastnäsite was found only at 80 °C and only in the La solution experiments and is the most insoluble and thermodynamically stable REE-carbonate phase. The mineral showed triangular-shaped shapes but no spherulitic morphology. The saturation index for this phase was calculated using the solubility products of the analogues (Nd)-kozoite and (Nd)-hydroxylbastnäsite (log*K*_sp_ = −22.3 ± 0.2 and −23.8 ± 0.1, respectively^73^). When (Nd)-kozoite is in equilibrium with the aqueous solution, the saturation index for (Nd)-hydroxylbastnäsite is SI = 1.50. This value is not high enough to promote crystallization via spherulitic growth, so we suggest then that the kozoite - hydroxylbastnäsite transformation process occurs via a dissolution-recrystallization mechanism in line with Vallina et al.^13^

In the Ce mixing solution experiments, cerianite was identified as the most thermodynamically stable phase as it is more insoluble (log*K*_sp_ = −59.3 ± 0.3 in 0.01 M NaClO_4_^94^) compared to the hydroxylbastnäsite (log*K*_sp_ = −23.8 ± 0.1^73^). This data is in agreement with the previous studies of Szucs et al.^16^ and Janos et al.^95^ The crystallization of cerianite triggers the decarbonation of the (Ce)-carbonates because of the pH-dependent redox oxidation from Ce^3+^ to Ce^4+^. This happens since Ce^4+^ has a stable electronic configuration of the nearest noble gas and is more stable than Ce^0^ and Ce^3+^.^96,97^ The initial pH after the formation of carbonate solids is ∼8.0–8.2, basic enough to promote the oxidation of Ce^3+^ to Ce^4+^ in solution.^16^ This is then directly translated into the formation of cerianite via dissolution–recrystallization of the Ce^3+^-carbonates.^16^ Cerianite crystals obtained from our experiments consisted of octahedral prisms with dimensions ranging from hundreds of nm to a few micrometers (Figure 9), sometimes forming spheroidal nanoaggregates (Figure 8h) which do not exhibit any preferred orientation, as the crystals are randomly oriented. Although the (Ce)-kozoite solubility product is unknown, the saturation index for cerianite when the aqueous solution is in equilibrium with (Ce)-lanthanite is 7.63, a value perfectly consistent with spherulitic growth.

## Conclusions

This study provides new insights into the crystallization pathways, mechanisms, kinetics, and energetics of (La)-, (Ce)-, (Pr)-, and (Nd)-carbonates formation at temperatures ranging from environmental to low hydrothermal conditions. Our findings reveal that the crystallization pathways of light REE-carbonates are more complex than previously thought, involving multiple intermediate phases and reversible reactions, especially in the early stages of their formation process. This complexity arises from the intricate interplay of various parameters: temperature, solution concentration, nanophase surface energy, and the ionic potential of the REE present in the aqueous solution.

The ionic potentials of different REEs significantly influence the structure of metastable phases and the crystallization pathways toward the thermodynamically stable end phase. For instance, a notable reversible hydration-dehydration reaction between lanthanite and tengerite occurs in the La and Ce systems at ambient temperature, indicating an atomic- and nanoscale rearrangement of the involved phases.

This in-depth knowledge of REE-carbonate crystallization mechanisms and pathways is fundamental for enhancing their potential applications in the material sciences industry and for the design of more efficient sequential extraction methods for REE via fractional crystallization.

## Supporting Information Available

Detailed view of the first stages of the crystallization reaction of (Ce)-lanthanite at 19 °C; Normalized turbidity graphs showing the evolution of the crystallization curve for (La)-, (Ce)-, (Pr)- and (Nd)-lanthanite at 19 °C; Avrami plots for (La)-lanthanite crystallization at different temperatures (19, 25, 30, and 40 °C); Avrami plots for (Ce)-lanthanite crystallization at different temperatures (19, 25, 30, and 40 °C); Avrami plots for (Pr)-lanthanite crystallization at different temperatures (19, 25, 30, and 35 °C); Avrami plots for (Nd)-lanthanite crystallization at different temperatures (19, 25, 45, and 55 °C); and crystallite size of lanthanite and tengerite during the reversible transformation (PDF)
