# Supplementary material for: Mechanistic Insights into the Early-Stage Crystallization and Nanophase Formation of Metastable Light Rare-Earth Carbonates
Source: Cryst Growth Des. 2025 Feb 8;25(4):945–62. doi: 10.1021/acs.cgd.4c01168 (PMC11843599; doi:10.1021/acs.cgd.4c01168)
Supplement: Supplementary file 1 — cg4c01168_si_001.pdf [file cg4c01168_si_001.pdf]

SUPPORTING INFORMATION FOR THE PAPER

Mechanistic insights into the early-stage crystallisation and nanophase formation of metastable light rare earth carbonates

Luca Terribili[a*], Adrienn Maria Szucs[a], Melanie Maddin[a], Kristina Petra Zubovic[a], Remi Rateau[a], Juan Diego Rodriguez-Blanco[a,b]

* terribil@tcd.ie

a Department of Geology. School of Natural Sciences. Trinity College Dublin, College green, Dublin D02PN40, Ireland.

b iCRAG. Department of Geology. School of Natural Sciences. Trinity College Dublin, College green, Dublin D02PN40, Ireland

Fig. SI-1. Detailed view of the very first stages of the crystallisation reaction of (Ce)-lanthanite at 19°C. It is possible to appreciate the initial sudden increase taking place a t ~ 0-10 s and the following sluggish phase at t~ 10-650 s before the main increase of the turbidity indicating the lanthanite crystallisation. The behaviour showed in this specific experiments can be taken as a model as it is consistent in all the other Uv-Vis experiments carried out.

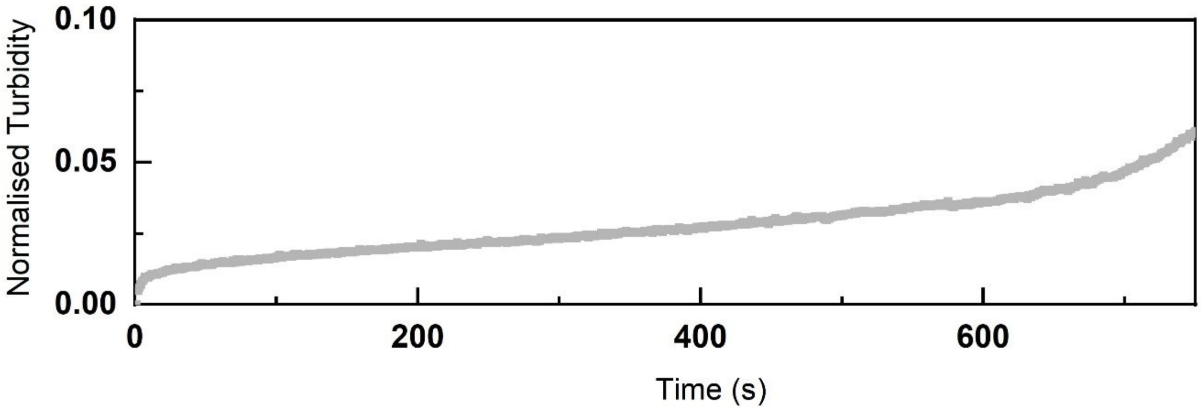

Fig. SI-2. Normalised turbidity graphs showing the evolution of the crystallisation curve for (La)-, (Ce)-, (Pr)- and (Nd)-lanthanite at 19 °C.

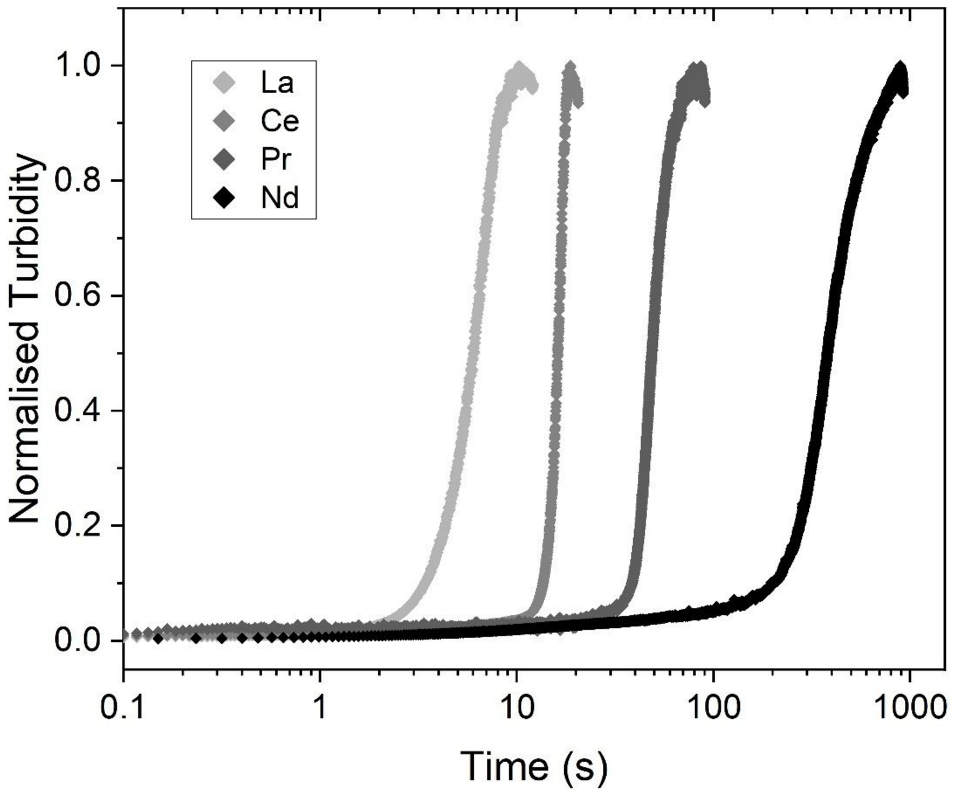

Fig. SI-3. Avrami plots for (La)-lanthanite crystallisation at different temperatures (19, 25, 30 and 40 °C).

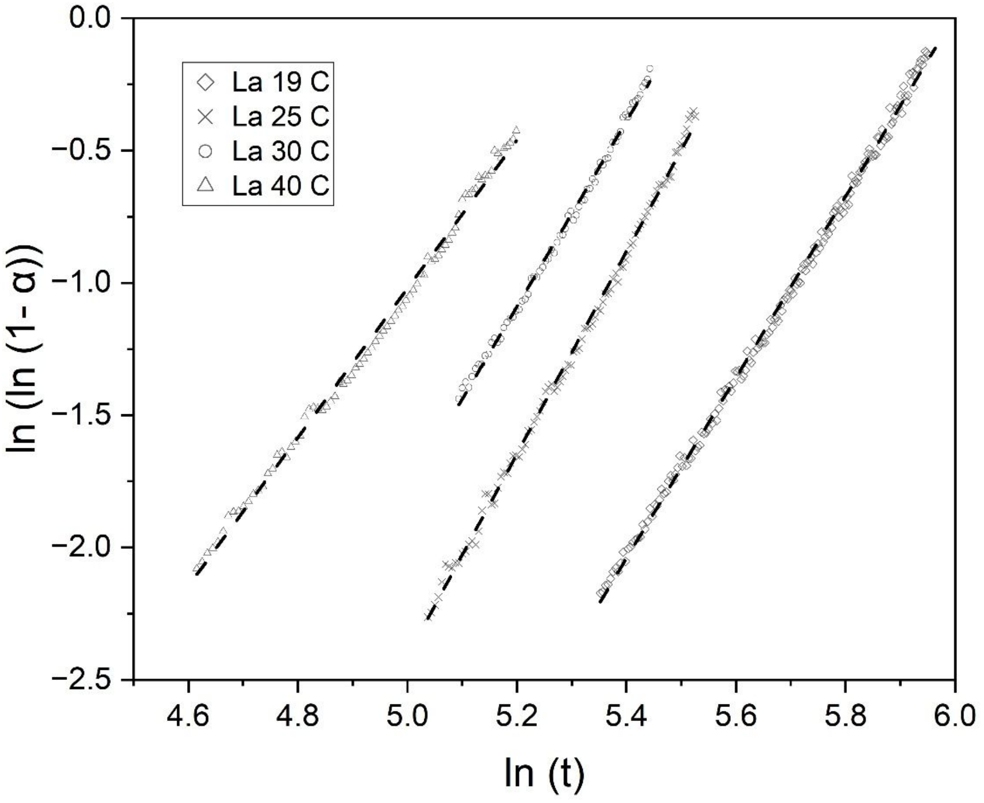

Fig. SI-4. Avrami plots for (Ce)-lanthanite crystallisation at different temperatures (19, 25, 30 and 40 °C).

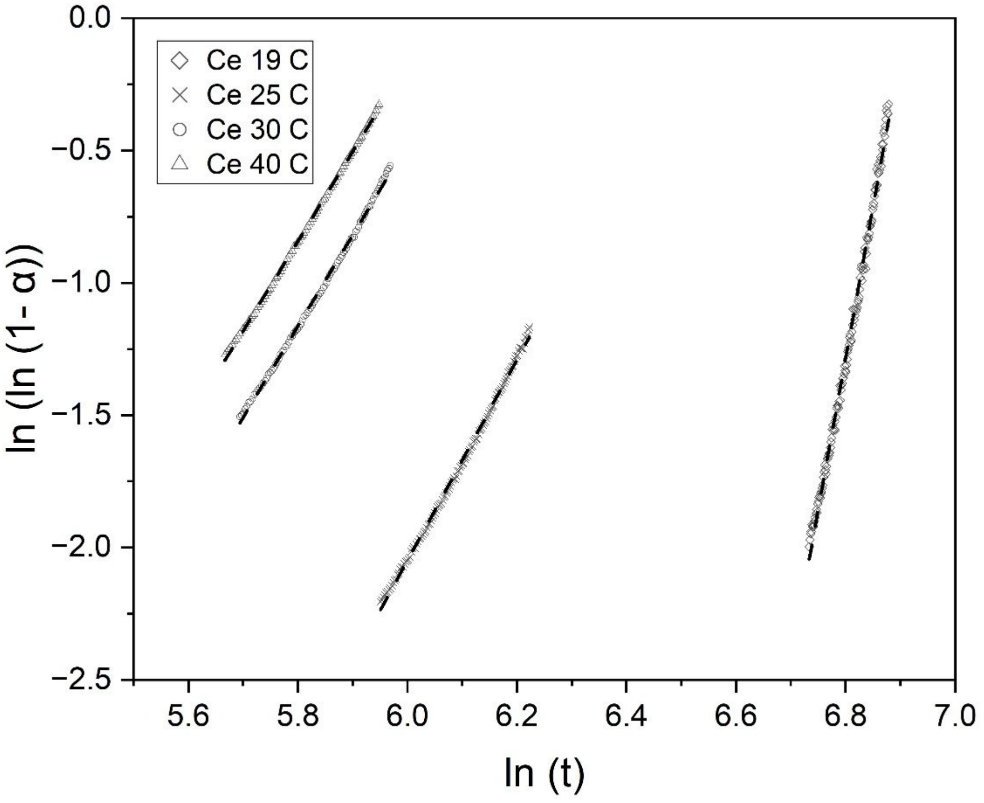

Fig. SI-5. Avrami plots for (Pr)-lanthanite crystallisation at different temperatures (19, 25, 30 and 35 °C).

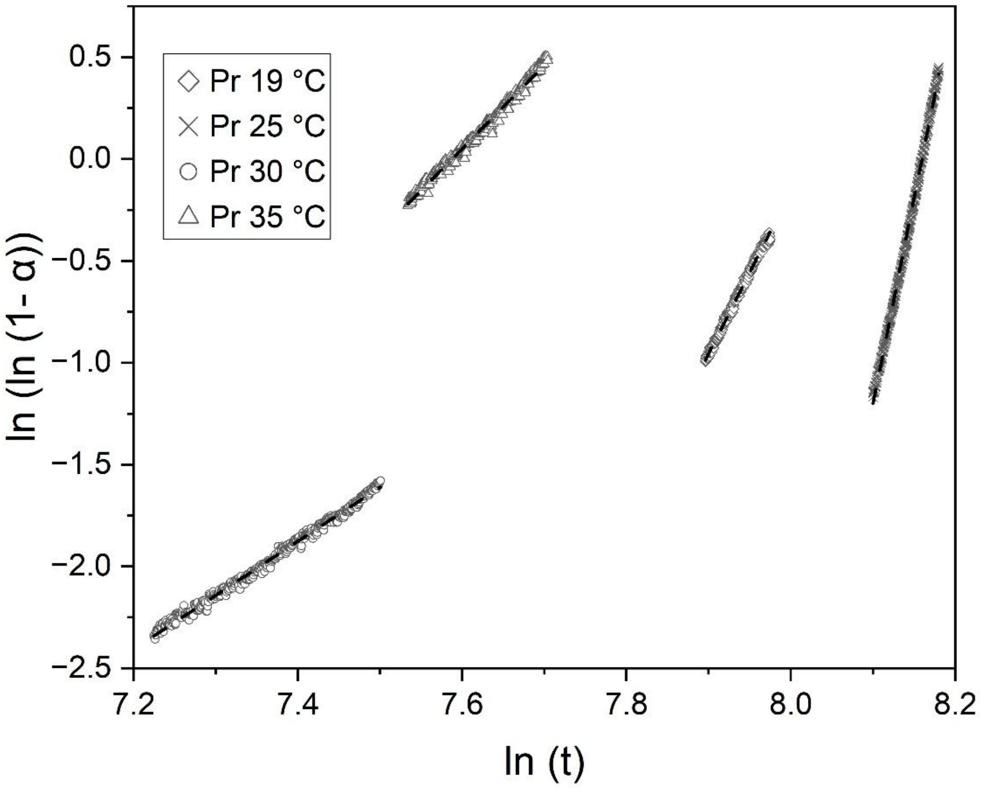

Fig. SI-6. Avrami plots for (Nd)-lanthanite crystallisation at different temperatures (19, 25, 45 and 55 °C).

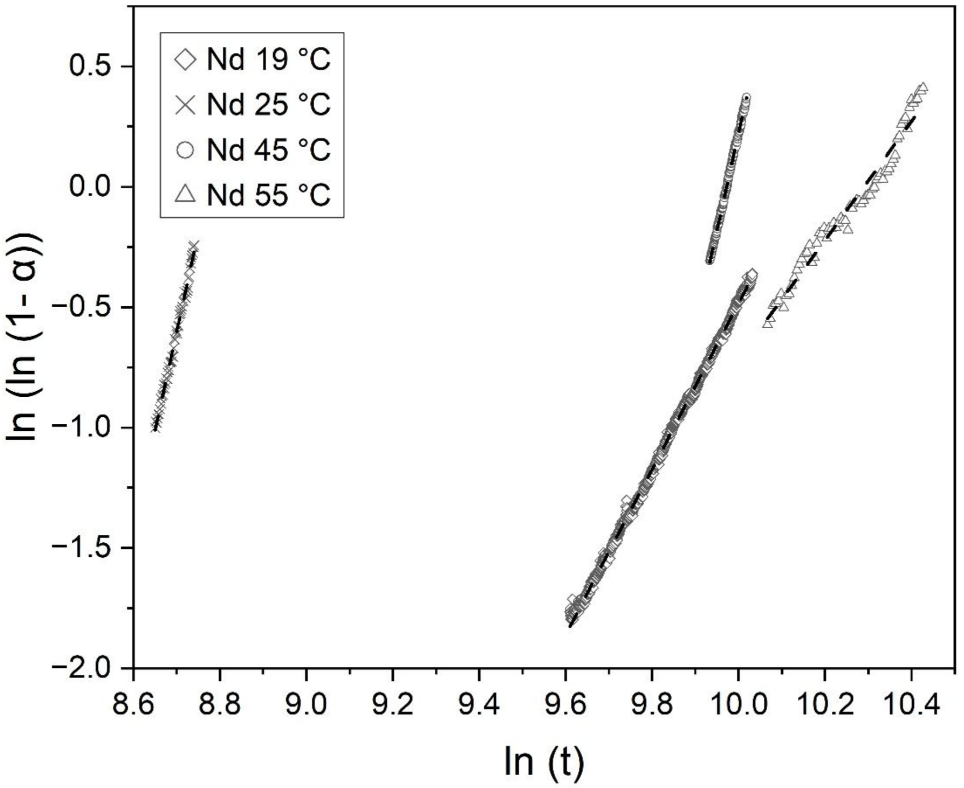

Table SI-1. Crystallite size of lanthanite and tengerite during the reversible transformation.

**Dehydration**				
	**Lanthanite**		**Tengerite**	
**Time (hours)**	**FWHM (º)**	**Crystallite size (Å)**	**FWHM (º)**	**Crystallite size (Å)**
0	0.100	883	/	/
5.5	0.062	1421	1.047	85
7	/	/	1.026	87
**Rehydration**				
	**Lanthanite**		**Tengerite**	
**Time (hours)**	**FWHM (º)**	**Crystallite size (Å)**	**FWHM (º)**	**Crystallite size (Å)**
0	/	/	1.026	87
5	0.051	1725	1.055	84
17.5	0.098	905	1.056	84
48.5	0.084	1053	0.991	90
57.5	0.087	1025	0.992	90
120.5	0.085	1040	0.897	99
